# The VCPIP1‐UBE3A Deubiquitination‐Ubiquitination Cascade Contributes to Castration‐Resistant Prostate Cancer Progression

**DOI:** 10.1002/advs.78433

**Published:** 2026-10-25

**Authors:** Fei Lin, Wen‐Cai Zheng, Yi‐Qiu Wang, Bin Lin, Yun‐Zhi Lin, Dong‐Ning Chen, Xiao‐Dong Li, Xue‐Yi Xue, Yong Wei, Ning Xu

**Affiliations:** ^1^ Department of Urology Urology Research Institute the First Affiliated Hospital Fujian Medical University Fuzhou China; ^2^ Department of Urology National Regional Medical Center Binhai Campus of the First Affiliated Hospital Fujian Medical University Fuzhou China; ^3^ Fujian Key Laboratory of Tumor Immunotherapy the First Affiliated Hospital Fujian Medical University Fuzhou China

**Keywords:** CRPC progression, deubiquitinases, prostate cancer, ubiquitination

## Abstract

The molecular mechanisms underlying progression from metastatic hormone‐sensitive prostate cancer (mHSPC) to metastatic castration‐resistant prostate cancer (mCRPC) remain incompletely understood. Paired proteomic profiling of specimens from 10 patients identified VCPIP1 as a candidate regulator, and its clinical relevance was evaluated in an independent cohort of 128 mHSPC patients. Elevated VCPIP1 expression was independently associated with shorter CRPC‐free survival. Functional studies demonstrated that VCPIP1 promoted prostate cancer proliferation, migration, invasion, and metastasis in a deubiquitinase activity‐dependent manner. Mechanistically, VCPIP1 directly bound UBE3A and stabilized it by removing K48‐linked polyubiquitin chains at Lys711, thereby enhancing UBE3A‐mediated p53 ubiquitination and proteasomal degradation. Wild‐type UBE3A, but not its catalytically inactive C833A mutant, rescued the malignant phenotypes suppressed by VCPIP1 depletion. TP53 knockdown partially reversed these effects in TP53‐wild‐type LNCaP cells but not in TP53‐mutant DU145 cells, indicating a TP53 context‐dependent contribution of p53. VCPIP1 depletion also reduced androgen receptor expression in LNCaP cells and enhanced enzalutamide sensitivity in patient‐derived organoids. These findings identify a VCPIP1/UBE3A deubiquitination–ubiquitination cascade contributing to CRPC progression and support further investigation of VCPIP1 as a potential prognostic biomarker and therapeutic target.

## Graphical abstract

VCIPIP1 emerges as a molecular link between protein stability and prostate cancer progression. By deubiquitinating and stabilizing UBE3A, VCIPIP1 promotes p53 degradation and progression to castration‐resistant prostate cancer. Elevated VCIPIP1 expression is associated with shorter castration‐resistant prostate cancer–free survival, highlighting the VCIPIP1–UBE3A–p53 axis as a potential target for delaying disease progression.

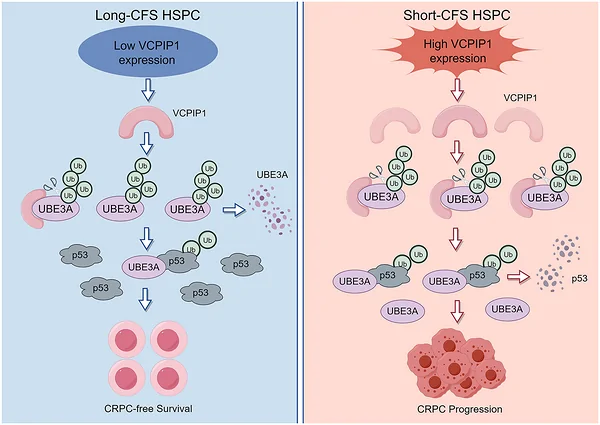

## 1 Introduction

Prostate cancer (PCa) is the most prevalent malignant neoplasm of the male genitourinary system, with its incidence rate exhibiting a consistent upward trend on a global scale [1]. In cases of localized prostate cancer, radical prostatectomy or radiotherapy typically results in favorable prognoses [2, 3]. However, a subset of patients was diagnosed at advanced stages, where metastatic prostate cancer presents substantial therapeutic challenges in clinical practice [4]. Androgen deprivation therapy (ADT) is the foundational treatment for advanced prostate cancer. Metastatic hormone‐sensitive prostate cancer (mHSPC) is characterized by metastatic prostate cancer that remains responsive to endocrine therapy [2]. In clinical settings, the majority of patients with treatment‐naïve metastatic prostate cancer fulfill the diagnostic criteria for mHSPC [5]. Recent advancements in the treatment paradigms for mHSPC have been groundbreaking. Novel combination therapies that integrate ADT with next‐generation androgen receptor pathway inhibitors or chemotherapy have significantly improved survival outcomes for mHSPC patients [6].

Nevertheless, the majority of prostate cancer cases inevitably advance to metastatic castration‐resistant prostate cancer (mCRPC) following ADT [7]. This stage is distinguished by significantly heightened malignant degree, accelerated disease progression despite the application of multimodal therapeutic approaches, and a notably poor prognosis [8]. Recent evidence indicated that an intensive triple combination therapy, comprising ADT, novel endocrine therapy, and docetaxel, may enhance outcomes in patients with metastatic prostate cancer [9, 10]. However, these intensified treatment regimens were linked to a substantial increase in adverse effects, and their long‐term efficacy necessitates validation through prolonged follow‐up studies [11]. For patients exhibiting low‐risk progression to mCRPC, sustained disease control can be effectively achieved through ADT in conjunction with endocrine therapy alone [12]. Over‐intensification of treatment may result in iatrogenic harm and unnecessary utilization of healthcare resources [13]. Consequently, identifying the critical molecular drivers of malignant transformation from mHSPC to mCRPC, as well as developing precision screening systems for rapidly progressing mHSPC subtypes, holds substantial clinical importance. This approach could facilitate the implementation of risk‐stratified therapies and optimize health economic outcomes.

In this study, paired prostate biopsy specimens were obtained from ten patients diagnosed with mHSPC at the initial diagnosis and upon progression to mCRPC for proteomic analysis. By integrating these findings with clinicopathological data, the deubiquitinating enzyme (DUB) VCPIP1 was identified as a pivotal protein associated with mCRPC progression. We conducted further investigations into the clinical significance and biological functions of VCPIP1 in prostate cancer, elucidating its role and underlying mechanisms in the malignant progression of mCRPC. This study identified VCPIP1 as a novel regulatory factor mediating the aggressive progression of prostate cancer, offering new insights for accurately identifying mHSPC patients with rapid clinical progression and facilitating personalized precision therapy to enhance patient outcomes.

## 2 Materials and Methods

### 2.1 Cell Culture

DU145 cells (RRID: CVCL_0105), LNCaP cells (RRID: CVCL_0395), RWPE‐1 cells (RRID: CVCL_3791), and HEK‐293T cells (RRID: CVCL_0063) were obtained from Procell Life Science & Technology Co., Ltd. (Wuhan, China). LNCaP cells were maintained in RPMI‐1640 medium supplemented with 10% fetal bovine serum (FBS). DU145 and HEK‐293T cells were cultured in MEM and DMEM media, respectively, each supplemented with 10% FBS. RWPE‐1 cells were grown in Keratinocyte Serum‐Free Medium. All cell lines were incubated at 37°C in a 5% CO_2_ atmosphere.

### 2.2 Clinical Specimens

This study involved the collection of paraffin‐embedded prostate biopsy specimens from 128 patients newly diagnosed with mHSPC who received ADT in combination with novel hormonal therapy (NHT). The clinicopathological characteristics of these patients are summarized in Table 1. Expert pathologists conducted histological examinations of the paraffin sections to verify the pathological status of all specimens. The primary endpoint was castration‐resistant prostate cancer‐free survival (CFS), defined as the duration from initial diagnosis to the progression to mCRPC. Patients exhibiting a CRPC‐free survival duration of six months or less were classified as having rapidly progressing mHSPC. The 6‐month cutoff was selected based on previous studies demonstrating that progression to castration resistance within 6 months is associated with particularly unfavorable clinical outcomes and has been used to define early progression in mHSPC [14, 15, 16]. Additionally, a matched‐pair sample proteomic analysis was performed, involving the collection of prostate biopsy specimens from 10 metastatic prostate cancer patients treated in our department. These samples were collected at both the initial mHSPC diagnosis and subsequent mCRPC progression stages. The clinicopathological characteristics of the 10 patients included in the paired proteomic cohort are summarized in Table S1. Three additional fresh biopsy specimens were collected from patients with metastatic prostate cancer following prostate‐specific membrane antigen‐positron emission tomography (PSMA‐PET)/ultrasound fusion‐guided targeted biopsy, with the aim of establishing patient‐derived organoid (PDO) models. Furthermore, three cases of newly resected postoperative prostate cancer tissues were acquired to establish a patient‐derived xenograft (PDX) model. Informed written consent was obtained from all participants, and the study was approved by the Ethics Committee of the First Affiliated Hospital of Fujian Medical University.

**TABLE 1:** Clinicopathological characteristics of 128 mHSPC patients.

Clinicopathological characteristics	N = 128
Age (years)	67(63–73)
<65	44
≥65	84
BMI (kg/m^2^)	23.41±2.61
≤25	90
>25	38
Diabetes mellitus	
Negative	106
Positive	22
ECOG PS	
0	87
1	41
PSA (ng/ml)	23.2(16.45–62.6)
<20	44
≥20	84
Disease volume	
Low volume	32
High volume	96
Visceral metastasis	
Negative	102
Positive	26
Gleason score	
<8	36
≥8	92
ALP≥ULN	
Negative	74
Positive	54

### 2.3 Patient‐Derived Organoid Models

Patient‐derived prostate cancer tissues were processed to generate organoid models. Initially, these tissues underwent enzymatic digestion to dissociate them into single cells and small clusters. The resultant cell suspension was then embedded in Matrigel (356231, Corning) and cultured in a 24‐well plate. Organoids were deemed appropriate for cytotoxicity assays once they attained a diameter of approximately 100 µm. The size and morphology of the organoids were assessed using microscopy. The enzalutamide was subsequently subjected to serial dilution, ranging from 10 to 0.0032 µM. Following a 96‐h incubation period, cell viability was evaluated utilizing the 3D CellTiter‐Glo assay (Promega, USA). The half‐maximal inhibitory concentration (IC50) values were calculated using GraphPad Prism 10.0 software (GraphPad Software, USA).

### 2.4 Quantitative Reverse‐Transcription Polymerase Chain Reaction (qRT‐PCR)

Total RNA was extracted utilizing the FastPure Cell/Tissue Total RNA Isolation Kit V2 (RC112‐01, Vazyme). Reverse transcription reactions were conducted in accordance with the protocol provided by the TransScript Green One‐Step qRT‐PCR SuperMix (TransGen Biotech, Beijing, China). Subsequently, quantitative real‐time PCR was performed using the Taq Pro Universal SYBR qPCR Master Mix (Vazyme) on the Step One PlusTM PCR System (Applied Biosystems; RRID: SCR_015805) to assess the relative mRNA expression levels. GAPDH expression was used as an internal reference for data analysis. The primer sequences employed are detailed in Table S2.

### 2.5 Plasmids Transfection and Lentivirus Transduction

The overexpression and mutant plasmids were procured from Shanghai GenePharma Co., Ltd (Shanghai, China). The Flag‐VCPIP1 and Myc‐UBE3A constructs were inserted into the Ubi‐MCS‐SV40‐puromycin vector by GENECHEM (Shanghai, China). Lentiviral vectors carrying short hairpin RNA (shRNA) targeting VCPIP1, UBE3A or TP53 were obtained from Shanghai GENECHEM Co., Ltd (Shanghai, China). Transfections were conducted using Lipofectamine 2000 (Invitrogen, Carlsbad, CA) following the manufacturer's instructions. Lentiviral infections of cells and organoids were performed in the presence of Polybrene. Subsequently, cells were cultured in a medium containing puromycin to select stable clones, 48 h post‐Polybrene treatment.

### 2.6 Immunofluorescence

Cells cultured on coverslips underwent fixation with 4% formaldehyde for 15 min, followed by permeabilization using 0.5% Triton X‐100 for 5 min. Subsequently, the cells were blocked with 1% bovine serum albumin (BSA) for 1 h and incubated overnight at 4°C with the indicated primary antibodies, including anti‐UBE3A (Proteintech; 10344‐1‐AP; RRID: AB_2211801), anti‐p53 (Santa Cruz Biotechnology; sc‐126; RRID: AB_628082), and anti‐VCPIP1 (Santa Cruz Biotechnology; sc‐531330; RRID: AB_3678493). After three washes with phosphate‐buffered saline (PBS), the cells were incubated with fluorophore‐conjugated secondary antibodies in the dark for 1 h. The cells were further stained with DAPI (Beyotime, China) for 10 min. Finally, the cells were visualized and imaged using a scanning microscope.

### 2.7 Immunohistochemistry (IHC) Staining

Briefly, the tissue sections were deparaffinized with xylene and rehydrated through a graded ethanol series after being heated at 60°C for 30 min. Endogenous peroxidase activity was inhibited using 3% hydrogen peroxide, and antigen retrieval was performed in a boiling citric acid buffer. To prevent non‐specific binding, normal sheep serum was applied as a blocking agent. The sections were then incubated overnight at 4°C with specific primary antibodies. On the following day, a secondary antibody was applied for 40 min. Visualization was achieved through Diaminobenzidine (DAB) staining (ZLI‐9017, ZSGB‐BIO, Beijing, China) for 2–3 min, followed by hematoxylin counterstaining for several minutes, differentiation for 2–5 s, and final mounting with neutral gum.

The final IHC score was determined using the formula: Immunoreactivity Score (IRS) = Intensity Score×Positive Score. The percentage of positively stained cells was classified into five categories: 0%–3% (score 0), 3%–25% (score 1), 26%–50% (score 2), 51%–75% (score 3), and 76%–100% (score 4). The staining intensity of positive cells was classified into four categories: no detectable staining (score 0), weak staining (score 1), moderate staining (score 2), and strong staining (score 3). The total IHC score ranged from 0 to 12, with scores of 4 or higher indicating high expression levels. All immunohistochemical sections were independently evaluated by two experienced pathologists who were blinded to the patients’ clinicopathological characteristics and clinical outcomes. Discrepant assessments were resolved by consensus. The antibodies utilized for the analyses included VCPIP1 (Proteintech; 17802‐1‐AP; RRID: AB_10699873), N‐cadherin (Proteintech; 22018‐1‐AP; RRID: AB_2813891), Vimentin (Proteintech; 10366‐1‐AP; RRID: AB_2273020), and Ki67 (Proteintech; 27309‐1‐AP; RRID: AB_2756525).

### 2.8 Western Blot Analysis, Co‐Immunoprecipitation (Co‐IP) and Antibodies

For the Western blot analysis, the samples were lysed using RIPA buffer (P0013B, Beyotime), and protein concentrations were determined with a BCA assay kit (P0010, Beyotime). Proteins were separated by electrophoresis on a 10% SDS‐PAGE gel according to their molecular weight and subsequently transferred onto PVDF membranes. The membranes were blocked and incubated overnight at 4°C with specific primary antibodies. Following a washing step, the membranes were incubated for 1 h at room temperature with HRP‐conjugated secondary IgGs. Visualization was performed using an ECL reagent (Advansta, USA).

For the Co‐IP assay, samples were lysed using an IP lysis buffer supplemented with a protease inhibitor cocktail, followed by centrifugation at 12 000 x g for 10 min. Subsequently, total protein concentrations were quantified utilizing a BCA kit. Equal amounts of lysate were then incubated with either IgG or specific primary antibodies in conjunction with protein A/G magnetic beads overnight at 4°C. The beads were subsequently washed three times with IP lysis buffer and prepared for subsequent western blot analysis.

The following antibodies were used in this study: anti‐VCPIP1 (CST; 88153; RRID: AB_2800115), anti‐UBE3A (Proteintech; 10344‐1‐AP; RRID: AB_2211801), anti‐β‐actin (Proteintech; 20536‐1‐AP; RRID: AB_10700003), anti‐p53 (Proteintech; 10442‐1‐AP; RRID: AB_2206609), anti‐AR (proteintech; 22089‐1‐AP; RRID:AB_11182176), anti‐polyubiquitin (CST; 58395; RRID: AB_3075532), anti‐K48 linkage‐specific polyubiquitin (CST; 8081S; RRID: AB_10859893), anti‐Flag (Sigma; F1804; RRID: AB_262044), anti‐Myc (Proteintech; 16286‐1‐AP; RRID: AB_11182162), anti‐HA (Proteintech; 51064‐2‐AP; RRID: AB_11042321), anti‐His (Proteintech; 10001‐0‐AP; RRID: AB_11232228).

### 2.9 Quantitative Proteomic Analyses

4D label free quantitative proteomics analyses were conducted by Jingjie PTM BioLabs (Hangzhou, China) employing liquid chromatography‐tandem mass spectrometry (LC‐MS/MS). In summary, following protein extraction and digestion, peptides were dissolved using liquid chromatography with mobile phase A and subsequently separated by a NanoElute ultra‐high‐performance liquid chromatography system. The peptides were further separated by this system and introduced into the capillary ion source for ionization, followed by analysis using timsTOF Pro mass spectrometry. The ion source voltage was maintained at 1.65 kV, and both the precursor ions of the peptide segments and their secondary fragments were detected and analyzed using high‐resolution TOF.

### 2.10 Identification of UBE3A Ubiquitination Modification Site

The identification of ubiquitination sites via mass spectrometry (MS) has been previously documented [17]. In summary, the UBE3A protein underwent fractionation through SDS‐PAGE, and the resulting protein bands were visualized using Coomassie blue staining. The gel lanes corresponding to the 100‐kDa region were excised and subjected to in‐gel trypsin digestion. Subsequently, the peptide samples were analyzed using mass spectrometry.

### 2.11 Surface Plasmon Resonance Assay

The direct interaction between VCPIP1 and UBE3A was evaluated using surface plasmon resonance (SPR) on a Biacore 1K system (Cytiva). UBE3A was immobilized on a Series S Sensor Chip CM5 by amine coupling. Serially diluted VCPIP1 protein (7.8–1000 nM) was injected at a flow rate of 30 µL/min at 25°C, with an association time of 60 s and a dissociation time of 180 s. Sensorgrams were analyzed using Biacore Insight Evaluation Software. Binding affinity was determined using kinetic fitting, and steady‐state affinity analysis was additionally performed to confirm the binding affinity.

### 2.12 Colony Formation Assay and EdU Staining Assay

For the colony formation assay, cells were seeded into six‐well plates at a density of 1×10^3^cells per well and incubated for a duration of two weeks. Post‐incubation, the cells were fixed using 4% formaldehyde and subsequently stained with 0.5% crystal violet. The resulting colonies were quantified utilizing microscopic examination. The EdU staining assay was performed employing an EdU kit (Beyotime). Cells were exposed to an EdU working solution at a concentration of 10 µM for 2 h. Following this, the cells were fixed in 4% paraformaldehyde for 15 min, permeabilized with 0.3% Triton X‐100 for an additional 15 min at ambient temperature, and washed with 3% BSA. The cells were then treated with a click reaction mixture for 30 min in the absence of light, followed by a 10‐min incubation with Hoechst 33342. Fluorescent images were subsequently acquired using a microscope.

### 2.13 Wound‐Healing Assay and Transwell Invasion Assay

In the wound‐healing assay, cells were seeded in 6‐well plates at a density of 5 × 10^5^ cells per well to form confluent monolayers. A sterile 200 µl pipette tip was then employed to manually introduce uniform wounds into the cell monolayers. The wounds were documented and quantified at 0 and 24 h following incubation in serum‐free medium. For the transwell invasion assay, cells were maintained in serum‐free medium for 24 h before being seeded into the upper chamber of transwell inserts at a density of 4 × 10^4^ cells per 200 µL per well. Prior to the experiment, the transwell inserts were coated with 20 µg of Matrigel. The lower chamber of the transwell plates was filled with 500 µL of medium containing 30% FBS. After 48 h of incubation at 37°C, noninvading cells were removed using a cotton swab, while invading cells were fixed with 4% paraformaldehyde and stained with 0.1% crystal violet. Subsequently, five random fields were selected and imaged using an inverted microscope.

### 2.14 In Vivo Tumorigenesis Assay

The animal experiments were conducted in accordance with the ethical guidelines approved by the Animal Experimentation Ethics Committee of Fujian Medical University. BALB/c nude mice (BALB/c‐Nu; BALB/cNj‐Foxn1^nu/Gpt; GemPharmatech Co., Ltd.; strain number: D000521; RRID: IMSR_GPT:D000521) were purchased from Guangdong GemPharmatech Co., Ltd. (Guangdong, China). M‐NSG mice (NOD‐Prkdc^scid Il2rg^em1/Smoc; Shanghai Model Organisms Center, Inc.; catalog number: NM‐NSG‐001; RRID: IMSR_NM‐NSG‐001) were purchased from Shanghai Model Organisms Center, Inc. (Shanghai, China).

To investigate the biological functions of VCPIP1 and UBE3A in vivo, a subcutaneous xenograft model was established by implanting stable DU145 cells (5 × 10^6^ cells per mouse) (including VCPIP1 overexpressed cells and control cells, VCPIP1 knockdown cells and shNC cells, UBE3A overexpressed cells and control cells, UBE3A knockdown cells and shNC cells) into the flanks of nude mice (*n* = 5 mice per group). Tumor growth was monitored and measured every week. After a period of 28 days, the mice were euthanized for tumor excision, and tumor volumes were calculated using the formula: π/6×ab^2^ (where a represents length and b represents width, with a > b).

The luciferase‐tagged DU145 cells were generated through transduction with a lentiviral construct encoding luciferase gene. Following transduction, cells were subjected to selection in neomycin‐containing media for a period exceeding 10 days before being utilized in any experimental procedures. In the tail vein metastasis model, 2 × 10^6^ DU145 cells, which stably overexpressed either VCPIP1 or UBE3A along with luciferase, were administered via injection into the tail vein of nude mice (*n* = 6 mice per group). After a duration of 6 weeks, in vivo signal detection was performed by anesthetizing the mice with isoflurane and subsequently imaging them using an in vivo imaging system (IVIS) system (PerkinElmer; RRID: SCR_018621).

To establish the castration‐resistant subcutaneous cell‐derived xenograft (CDX) model, 8 × 10^6^ LNCaP cells were combined with Matrigel in a 1:1 ratio and injected into the flanks of BALB/c nude mice. For the PDX model, patient tissues were sectioned into 1 × 1 × 1 mm^3^ fragments, mixed with Matrigel in a 1:1 ratio, and subcutaneously implanted into M‐NSG mice. Tumor formation at the implantation site, reaching a volume exceeding 500 mm^3^, was deemed indicative of successful engraftment. In cases of successful engraftment, the mouse was anesthetized, and the primary tumor (F0) was excised for serial transplantation into subsequent generations (F1, F2). Typically, the F3 generation was expanded for further research purposes. Once the xenograft attained a volume of 500 mm^3^, the mice were randomly allocated into two groups: HSPC and CRPC. Mice in the CRPC group underwent surgical castration, while those in the HSPC group received a sham operation. Post‐surgery, the xenograft volume in the CRPC group initially decreased but subsequently exhibited rapid growth. Once the xenograft volume reached 1000 mm^3^, mice from both groups were euthanized, and their xenografts were harvested for further analysis.

To develop the castration‐resistant orthotopic xenograft model, 2 × 10^6^ luciferase‐tagged LNCaP cells, either stably overexpressing VCPIP1 or serving as controls, were implanted into the dorsal prostate of Balb/c nude mice. Orthotopic prostate tumorigenesis was monitored weekly via bioluminescence imaging using an IVIS Spectrum. Upon detection of orthotopic prostate tumors, the mice underwent surgical castration. Following these procedures, the models were monitored weekly for orthotopic prostate tumorigenesis and potential distant metastatic lesions through bioluminescence imaging using an IVIS Spectrum system. The metastatic progression following castration was interpreted as the transition of our HSPC models to CRPC.

### 2.15 Statistical Analysis

All statistical analyses were performed using GraphPad Prism version 8.0 and SPSS version 26.0. Quantitative data are presented as the mean ± standard deviation (SD), unless otherwise indicated. The exact number of independent biological replicates or animals used in each experiment is specified in the corresponding figure legend. For continuous variables, normality and homogeneity of variance were assessed before parametric testing. Comparisons between two independent groups were performed using an unpaired, two‐tailed Student's t‐test when the assumptions of normality and homogeneity of variance were satisfied. For comparisons involving more than two groups, one‐way analysis of variance (ANOVA) followed by an appropriate multiple‐comparison test was used. For experiments involving repeated measurements over time, two‐way repeated‐measures ANOVA was performed. When the assumptions for parametric testing were not satisfied, the Wilcoxon rank‐sum test or Kruskal–Wallis test was applied. CRPC‐free survival was estimated using the Kaplan–Meier method and compared using the log‐rank test. Cox proportional hazards regression analyses were performed to identify factors independently associated with CRPC‐free survival. Significance levels are denoted as follows: ^*^ *p* < 0.05; ^**^ *p* < 0.01; ^***^ *p* < 0.001, ^****^ *p* < 0.0001.

## 3 Results

### 3.1 Identification of Key Proteins in CRPC Progression Using 4D Label‐Free Proteomics

Prostate biopsy specimens were obtained from ten patients with mHSPC at initial diagnosis and during their progression to mCRPC for 4D label‐free proteomic analysis. The proteomic analysis identified a total of 1144 differentially expressed proteins (DEPs), with 808 proteins exhibiting upregulation and 336 proteins showing downregulation in mCRPC tissues (Figure 1A,B). Enrichment analysis of the Kyoto Encyclopedia of Genes and Genomes (KEGG; RRID: SCR_012773) signaling pathways associated with these differentially expressed proteins indicated their predominant involvement in pathways related to “nucleocytoplasmic transport,” “ribosome biogenesis in eukaryotes,” “ECM‐receptor interaction,” “valine, leucine and isoleucine degradation,” as well as “fatty acid biosynthesis and degradation” (Figure 1C).

**FIGURE 1:**
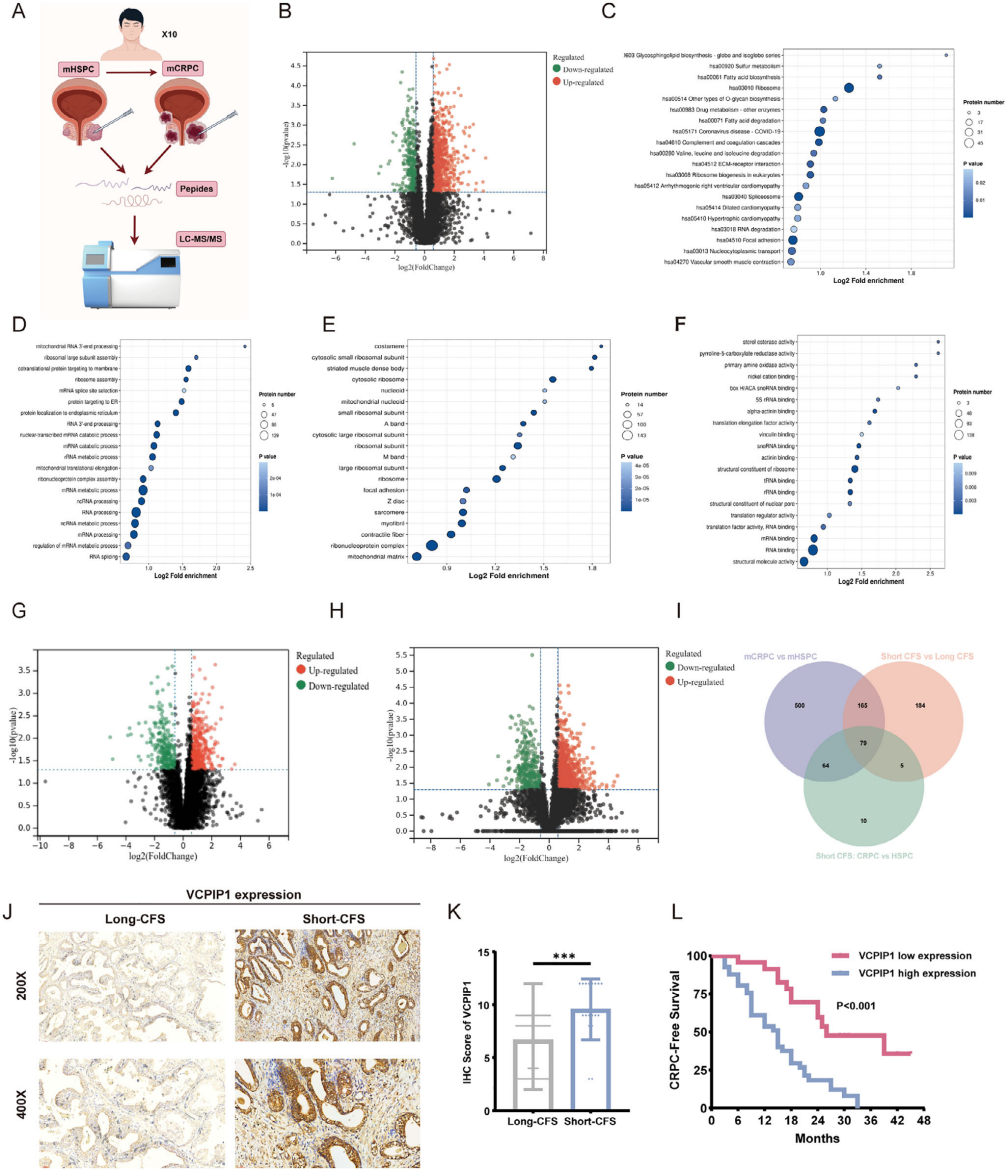
Identification of key differentially expressed proteins related to the progression of CRPC through 4D label‐free proteomic analysis. (A) Schematic overview of the experimental design showing proteomic profiling based on 10 paired HSPC/CRPC samples. (B) Volcano plot demonstrating differential protein expression patterns between HSPC and CRPC tissues. (C) KEGG pathway enrichment analysis of differentially expressed proteins (DEPs). Gene Ontology (GO) enrichment analysis of DEPs categorized into Biological Processes (D), Cellular Components (E), and Molecular Functions (F). (G) Comparative volcano plot depicting DEPs in HSPC tissues between patients with long CFS and those with short CFS. (H) Comparative volcano plot depicting DEPs between the paired HSPC/CRPC tissues in short‐CFS group. (I) Venn diagram demonstrating intersection of upregulated DEPs across the three comparative groups. (J,K) IHC staining of VCPIP1 expression based on CFS stratification. Differences in VCPIP1 expression between the short‐ and long‐CFS groups were analyzed using the Wilcoxon rank‐sum test. (L) Comparative Kaplan–Meier survival analysis of CFS between high and low VCPIP1 expression groups. ^***^ *p *< 0.001.

A Gene Ontology (GO; RRID: SCR_002811) analysis was conducted to elucidate the molecular characteristics and functional attributes of these DEPs from three distinct perspectives: biological processes, cellular components, and molecular functions. The analysis revealed significant enrichment of these proteins in biological processes such as “mRNA metabolic process,” “RNA processing,” “ribosome assembly,” and “RNA splicing.” In terms of cellular components, DEPs were predominantly associated with the “ribonucleoprotein complex,” “ribosomal subunit,” “mitochondrial matrix,” and “myofibril.” Concerning molecular functions, DEPs were primarily involved in “RNA binding,” “translation elongation factor activity,” “structural constituent of nuclear pore,” and “structural constituent of ribosome” (Figure 1D–F).

Follow‐up data from the 10 mHSPC patients were collected, and a subgroup analysis was performed based on the speed of disease progression. In comparison to the biopsy tissues obtained during the mHSPC stage in the long CFS group (CFS > 6 months), a total of 433 proteins were found to be upregulated in the biopsy tissues of the short CFS group (CFS ≤ 6 months) (Figure 1G). A paired‐sample analysis, which involved a longitudinal comparison of tissue samples before and after disease progression within the same patient, revealed that in the short CFS group, 158 proteins were upregulated in CRPC‐stage tissues compared to those in mHSPC‐stage biopsy tissues (Figure 1H). By intersecting the upregulated proteins identified in the three analyses, 79 overlapping upregulated proteins were identified (Figure 1I). The candidate proteins were subjected to further analysis based on their differential expression levels, potential involvement in tumor progression, regulatory functions, and novelty within the context of prostate cancer. Particular attention was given to enzymes implicated in post‐translational modifications, specifically deubiquitinating enzymes, due to the pivotal role that dysregulation of the ubiquitin‐proteasome system plays in cancer progression and therapeutic resistance [18]. Among the top 20 proteins exhibiting the highest fold‐change values when comparing mHSPC tissues from the short CFS group to the long CFS group, VCPIP1, a member of the ovarian tumor protease family of deubiquitinating enzymes, was identified. Although recent studies have demonstrated that VCPIP1 exhibits multifaceted regulatory functions in tumor development, its biological function in prostate cancer and its potential role in the transition from HSPC to CRPC remain unexplored [19, 20]. Consequently, VCPIP1 was selected for subsequent clinical and mechanistic investigations. The fold‐change rankings and relevant proteomic data for the top 20 upregulated proteins are provided in Table S3.

### 3.2 VCPIP1 Plays a Crucial Role in the Malignant Progression From mHSPC to mCRPC

To elucidate the clinical implications of VCPIP1 expression in mHSPC progression, IHC staining was conducted to evaluate VCPIP1 expression in prostate biopsy samples from a cohort of newly diagnosed mHSPC patients. The findings indicated that VCPIP1 expression was significantly higher in tumor tissues from patients in the short CFS group compared to those in the long CFS group (Figure 1J,K). Survival analysis further revealed that mHSPC patients with elevated VCPIP1 expression experienced a significantly diminished duration of CRPC‐free survival in comparison to those with lower expression levels (*p* < 0.001) (Figure 1L).

A comparative analysis of clinicopathological characteristics between the long and short CFS groups demonstrated that the short CFS group exhibited significantly elevated VCPIP1 expression levels (*p* = 0.018), a greater prevalence of abnormal serum alkaline phosphatase (ALP) (*p* = 0.037), and a higher incidence of visceral metastasis (*p* < 0.001) in comparison to the long CFS group (Table 2). These intergroup disparities further corroborate the more aggressive biological behavior of tumors observed in the short CFS group. The univariate analysis revealed that a prostate‐specific antigen (PSA) level of ≥20 ng/mL (*p *< 0.001; HR = 3.371), a Gleason score of ≥8 (*p *< 0.001; HR = 2.423;), high disease volume (*p* = 0.005; HR = 2.020), presence of visceral metastasis (*p *< 0.001; HR = 5.287), and elevated VCPIP1 expression (*p *< 0.001; HR = 3.660) were significantly correlated with reduced CFS in mHSPC patients. In subsequent multivariate Cox regression modeling, PSA levels of ≥20 ng/mL (*p* = 0.003; HR = 2.597), visceral metastasis (*p *< 0.001; HR = 4.547), and high VCPIP1 expression (*p *< 0.001; HR = 2.905) emerged as independent predictors of decreased CFS in this patient cohort (Table 3). These results suggested that VCPIP1 may play a pivotal role in the malignant progression from mHSPC to mCRPC.

**TABLE 2:** Comparison of clinicopathological characteristics of mHSPC patients between Long‐CFS and short‐CFS group.

Clinicopathological characteristics	Number of cases	CRPC‐Free Survival		*p* value
		Long (*n* = 110)	Short(*n* = 18)	
Age (years)				
<65	44	40	4	*p* = 0.294
≥65	84	70	14	
BMI (kg/m^2^)				
≤25	90	76	14	*p* = 0.583
>25	38	34	4	
Diabetes mellitus				
Negative	106	91	15	*p* = 0.950
Positive	22	19	3	
ECOG				
0	87	77	10	*p* = 0.277
1	41	33	8	
PSA (ng/ml)				
<20	44	40	4	*p* = 0.294
≥20	84	70	14	
Disease volume				
Low volume	32	30	2	*p* = 0.238
High volume	96	80	16	
Visceral metastasis				
Negative	102	98	4	*p *< 0.001^*^
Positive	26	12	14	
Gleason score				
<8	36	32	4	*p* = 0.778
≥8	92	78	14	
ALP≥ULN				
Negative	74	68	6	*p* = 0.037*
Positive	54	42	12	
VCPIP1 expression				
Low	46	44	2	*p* = 0.018*
High	82	66	16	

**TABLE 3:** The Univariate and multivariate Cox regression analyses of independent predictors for CFS in patients with mHSPC.

Clinical characters	Univariate analysis			Multivariate analysis		
	HR	95% CI	*p* value	HR	95% CI	*p* value
Age (years)						
<65 vs. ≥65	0.729	0.477–1.114	0.144	0.989	0.626–1.564	0.963
BMI (kg/m^2^)						
<25 vs. ≥25	1.160	0.753–1.787	0.500	1.244	0.771–2.006	0.371
Diabetes mellitus	0.639	0.354–1.154	0.138	0.684	0.362–1.291	0.241
ECOG						
0 vs. 1	0.696	0.443–1.094	0.116	0.797	0.5–1.271	0.340
PSA (ng/ml)						
<20 vs. ≥20	3.371	1995–5.697	**<0.001**	2.597	1.375–4.905	**0.003**
Disease volume						
low vs. high	2.020	1.238–3.296	**0.005**	0.683	0.360–1.297	0.244
Visceral metastasis	5.287	3.178–8.795	**<0.001**	4.547	2.583–8.005	**<0.001**
Gleason score						
<8 vs. ≥8	2.423	1.475–3.981	<0.001	1.111	0.589–2.094	0.745
ALP≥ULN	1.419	0.940–2.140	0.096	1.470	0.937–2.306	0.094
VCPIP1 expression						
low vs. high	3.660	2.257–5.935	**<0.001**	2.905	1.696–4.974	**<0.001**

### 3.3 VCPIP1 Promotes PCa Cell Proliferation, Migration and Invasion In Vitro and In Vivo

To examine the role of VCPIP1 in modulating the biological behavior of prostate cancer, initial baseline cellular analyses were conducted utilizing qRT‐PCR and Western blot techniques. The findings indicated a significant upregulation of both mRNA and protein expression levels of VCPIP1 in hormone‐sensitive prostate cancer cells (LNCaP) and castration‐resistant prostate cancer cells (DU145) when compared to normal prostate epithelial cells (Figures S1A‐B). Subsequently, stable cell lines with either knockdown or overexpression of VCPIP1 were successfully generated in DU145 and LNCaP prostate cancer cells via lentiviral transduction, with the alteration in VCPIP1 expression verified through qRT‐PCR and Western blot analysis (Figure S1C–J). The EdU assay indicated that the knockdown of VCPIP1 significantly suppressed the proliferation of DU145 and LNCaP cells, as illustrated in Figure 2A,B. This finding was further corroborated by colony formation assays, which demonstrated that VCPIP1 knockdown adversely affected the clonogenic efficiency of DU145 and LNCaP cells (Figure 2C,D). Additionally, transwell and wound‐healing assays suggested that diminished expression of VCPIP1 could impede the invasion and migration capacities of prostate cancer cells (Figure 2E–H).

**FIGURE 2:**
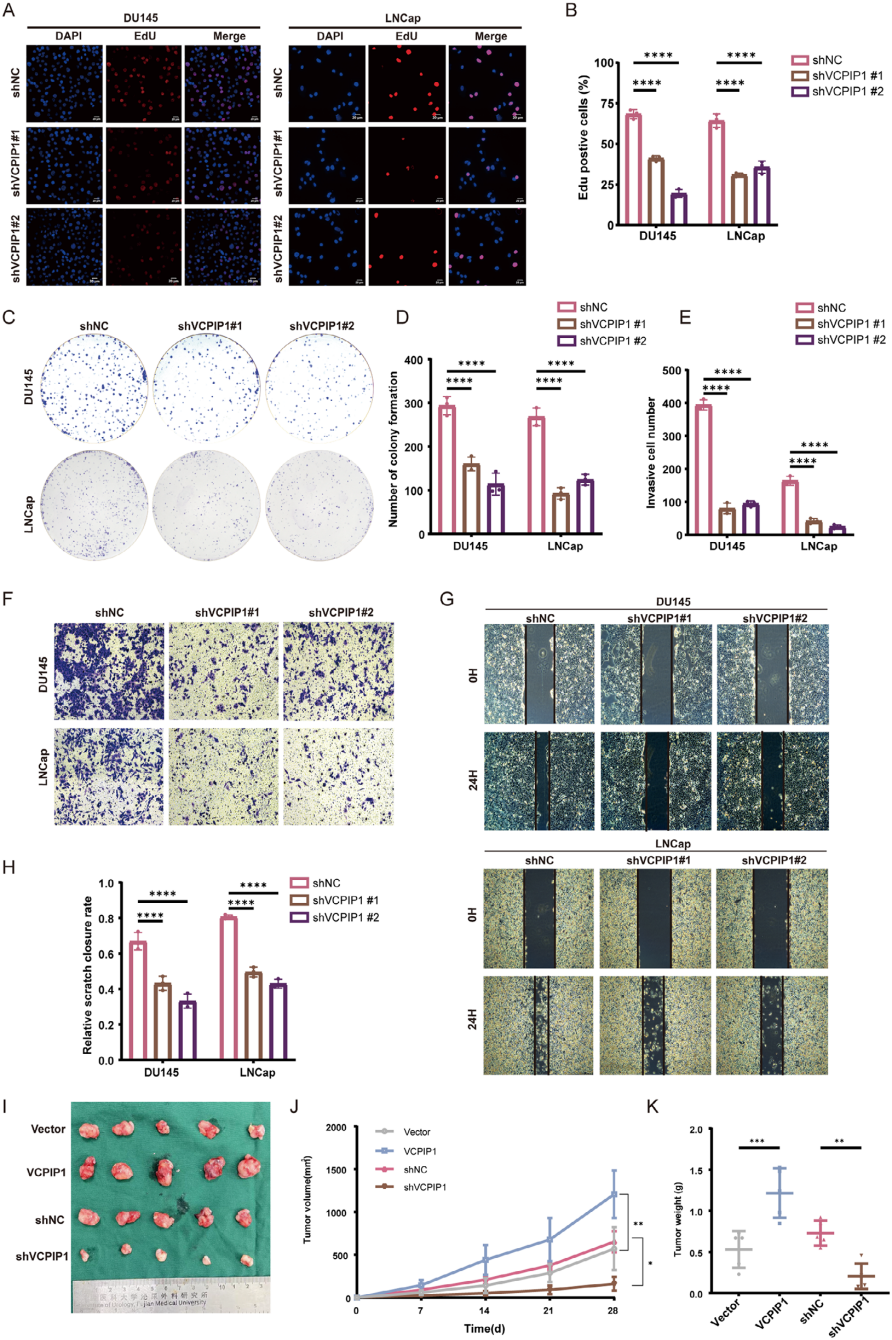
Knockdown of VCPIP1 suppressed PCa cell proliferation, migration, invasion, and tumor growth in vitro and in vivo. (A,B) The effect of knockdown of VCPIP1 on the proliferation of DU145 and LNCaP cells was detected by EdU assay. (C,D) The effect of knockdown of VCPIP1 on the colony forming ability of DU145 and LNCaP cells was detected by colony formation assay. (E,F) The effect of knockdown of VCPIP1 on the invasion ability of DU145 and LNCaP cells was detected by the Transwell assay. (G,H) The effect of knockdown of VCPIP1 on the migration ability of DU145 and LNCaP cells was detected by the scratch assay. (I–K) Different groups of DU145 cells (Vector, VCPIP1, shNC, and shVCPIP1) were subcutaneously inoculated in nude mice, *n* = 5. The tumor volume was measured every week, and the growth curves were drawn. The tumor volume was calculated according to the formula: V = π/6×length×width^2^; after tumor cells injection 28 days, the nude mice were sacrificed to measure tumor weight. Data are presented as the mean ± SD from three independent experiments. Statistical significance was assessed using one‐way ANOVA for panels B, D, E, H, and K, and two‐way repeated‐measures ANOVA for panel J. ^*^ *p *< 0.05, ^**^ *p *< 0.01, ^***^ *p *< 0.001, ^****^ *p *< 0.0001.

To investigate the impact of VCPIP1 on PCa tumor growth in vivo, we injected DU145 cells, either stably overexpressing or with knockdown of VCPIP1, into the subcutaneous axilla of nude mice to establish subcutaneous xenograft tumor models. The results indicated that the tumor growth rate, volume, and weight were significantly increased in the VCPIP1 overexpression group compared to the control group, whereas the VCPIP1 knockdown group exhibited a markedly inhibitory effect (Figure 2I–K). IHC staining demonstrated that the downregulation of VCPIP1 expression resulted in a simultaneous reduction in the mesenchymal phenotype markers, N‐cadherin and Vimentin, along with a significant decrease in the proportion of Ki67‐positive proliferative cells within the xenograft tumors (Figure 3A,B).

**FIGURE 3:**
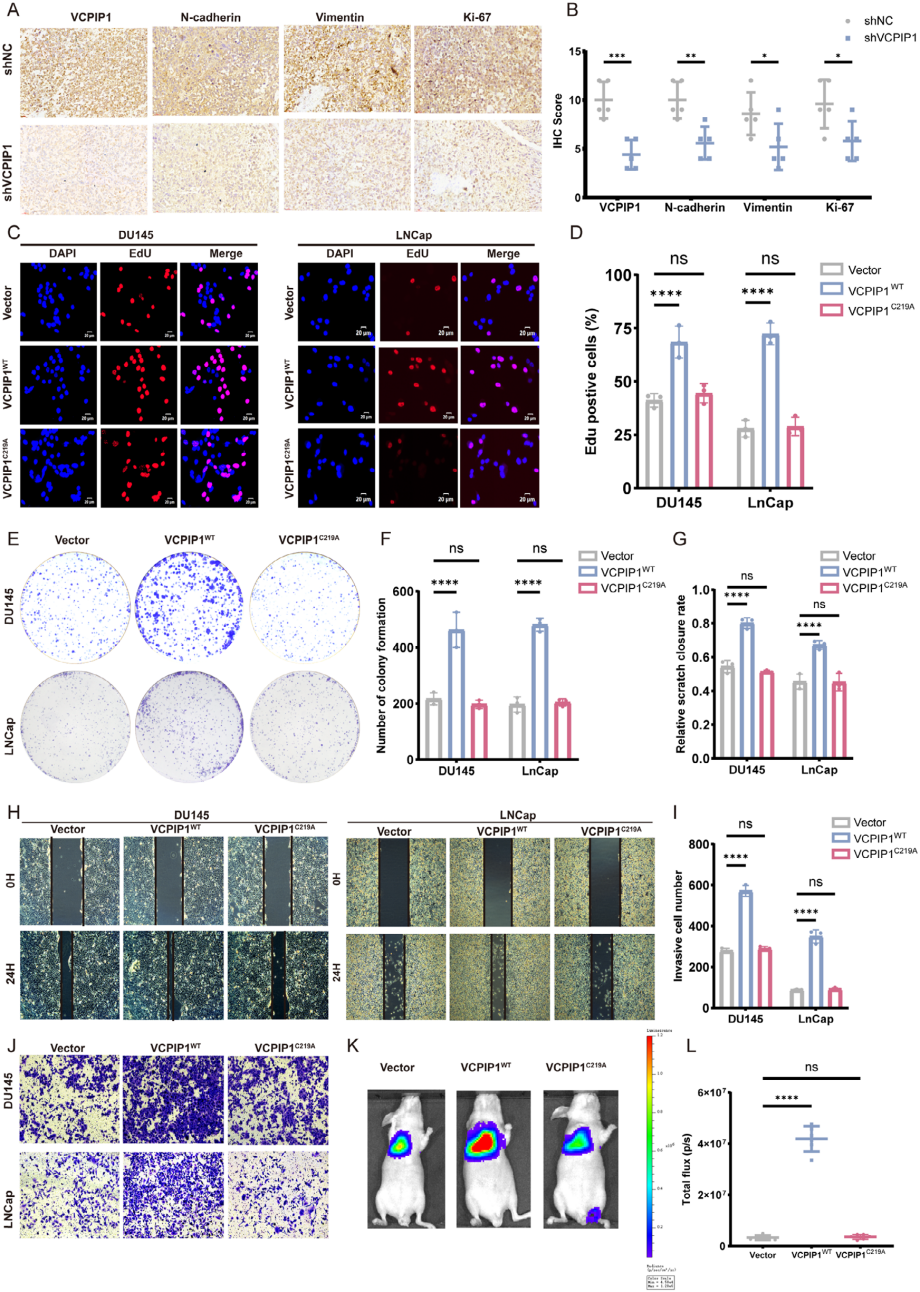
VCPIP1 promoted prostate cancer progression via its deubiquitinase activity. (A,B) IHC staining demonstrating the effects of VCPIP1 knockdown on epithelial‐mesenchymal transition (EMT)‐related markers and Ki67 proliferation index. Differences in immunohistochemical scores were analyzed using the Wilcoxon rank‐sum test. The proliferation rates of PCa cells were assessed using EdU incorporation (C,D) and colony formation assays (E,F) following transfection with either the vector, VCPIP1 wild‐type (WT), or VCPIP1‐C219A. (G,H) The migratory capacity of DU145 and LNCaP cells was determined through wound healing assays post‐transfection with the vector, VCPIP1 WT, or VCPIP1‐C219A. (I,J) The invasive potential of DU145 and LNCaP cells was evaluated using Transwell assays after transfection with the vector, VCPIP1 WT, or VCPIP1 C219A. (K,L) The metastatic potential of PCa cells was assessed through in vivo bioluminescence imaging subsequent to the tail vein injection of luciferase‐tagged DU145 cells, which were transfected with either the vector, VCPIP1 WT, or VCPIP1 C219A, *n* = 6. Data are presented as the mean ± SD from three independent experiments. Statistical significance was assessed using one‐way ANOVA for panels D, F, G, I, and L. ^*^ *p *< 0.05, ^**^ *p *< 0.01, ^***^ *p *< 0.001, ^****^ *p *< 0.0001.

### 3.4 VCPIP1 Promotes Prostate Cancer Progression via Its Deubiquitinase Activity

Given that VCPIP1 is a member of the deubiquitinase family, it is plausible that VCPIP1 modulates cellular biological behaviors through its deubiquitinase activity. Previous studies have identified the 219th cysteine as the catalytic residue of VCPIP1 [19]. To elucidate the underlying mechanisms, we sought to investigate the specific role of VCPIP1's catalytic activity. Consequently, we engineered a catalytically inactive variant of VCPIP1 (C219A) and subsequently assessed its impact on prostate cancer cells.

EdU incorporation assays showed that overexpression of VCPIP1 markedly promoted cell proliferation, whereas the catalytically inactive VCPIP1 mutant failed to produce a comparable effect (Figure 3C,D). Consistently, colony formation assays further demonstrated that VCPIP1 overexpression significantly enhanced the clonogenic capacity of PCa cells, while the mutant form exhibited minimal effects (Figure 3E,F). Wound‐healing assays confirmed that VCPIP1 overexpression accelerated PCa cell migration, whereas the mutant variant did not significantly alter migratory capacity (Figure 3G,H). Similarly, Transwell assays further demonstrated that VCPIP1 overexpression significantly enhanced the invasive abilities of PCa cells, while the mutant form exhibited little to no effect (Figure 3I,J). In addition, tail vein metastasis assays revealed that stable overexpression of VCPIP1 markedly increased metastatic colonization in vivo, as evidenced by stronger bioluminescence signals detected by IVIS imaging, whereas this pro‐metastatic effect was largely abolished in cells expressing the catalytic mutant (Figure 3K,L).

### 3.5 VCPIP1 Specifically Interacts With and Stabilizes UBE3A Protein

To further elucidate the molecular mechanisms by which VCPIP1 facilitated the progression of prostate cancer, Co‐IP coupled with mass spectrometry and proteomic analysis were conducted to identify potential substrate proteins of VCPIP1. The findings revealed that, in comparison to the control group, 449 proteins were upregulated, while 1047 proteins were downregulated following VCPIP1 knockdown (Figure 4A,B). Additionally, Co‐IP combined with mass spectrometry identified 1214 potential VCPIP1‐interacting proteins (Figure 4C). The candidate substrates were refined by intersecting 808 proteins that are upregulated in CRPC tissues with 1047 proteins that are downregulated following VCPIP1 knockdown, alongside 1214 potential VCPIP1‐interacting proteins identified through mass spectrometry. This integrated analysis yielded seven potential substrates of VCPIP1 (Figure 4D). Among these candidates, UBE3A exhibited the highest score in the Co‐IP/MS analysis and was therefore prioritized for subsequent validation and mechanistic investigation (Figure 4E,F).

**FIGURE 4:**
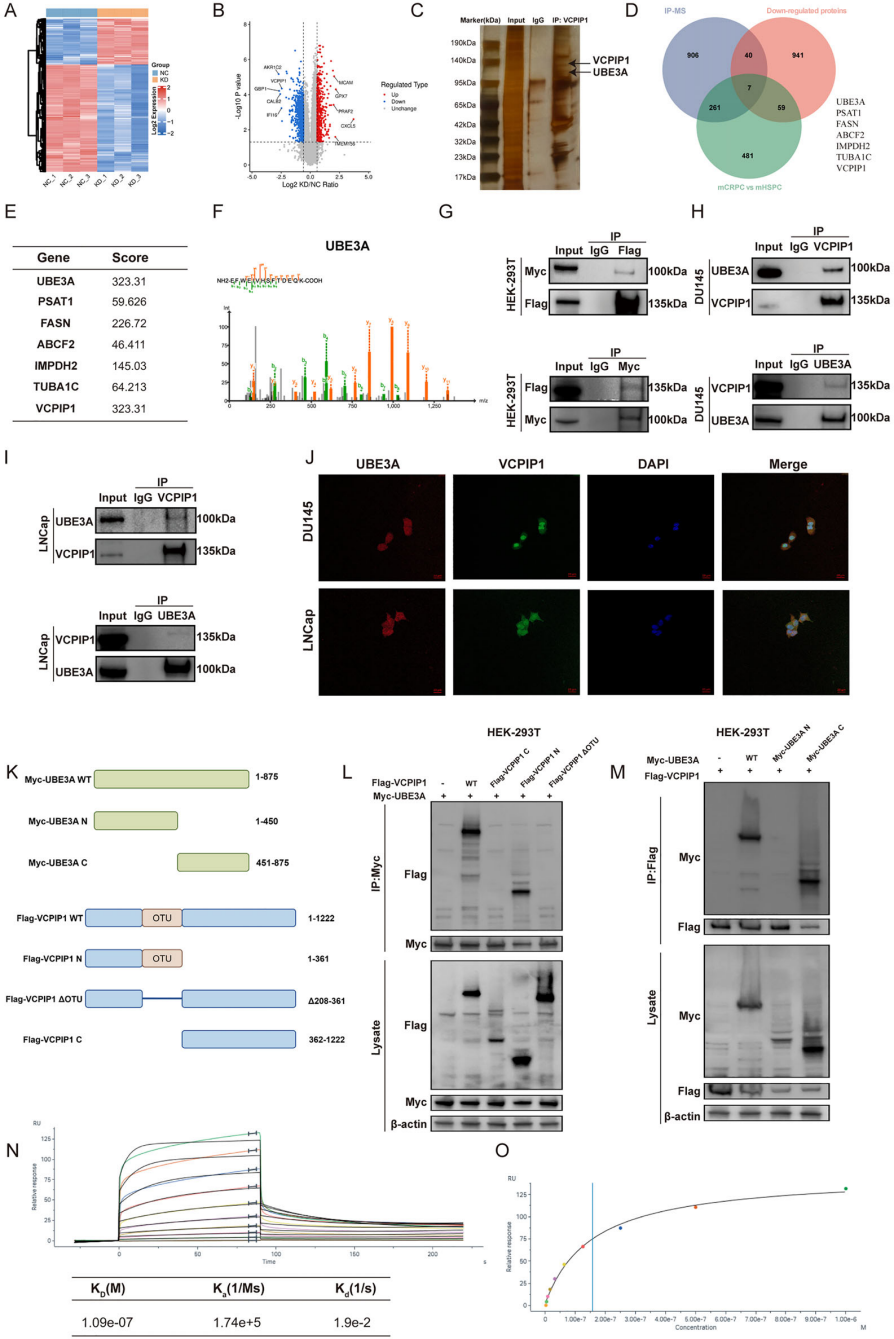
Identification of VCPIP1 candidate substrates. (A) Hierarchical clustering heatmap of proteomic profile of DU145 prostate cancer cells (shVCPIP1 vs. shNC). (B) Volcano plot of proteomic profile of DU145 prostate cancer cells (shVCPIP1 vs. shNC). (C) Silver staining showed the proteins bound to VCPIP1. (D) Venn diagram showed the intersection of proteins with upregulated expression in CRPC tissues, proteins with downregulated expression after VCPIP1 knockdown, and potential binding proteins of VCPIP1 identified by mass spectrometry. (E) Co‐IP/MS scores of the seven candidate VCPIP1‐interacting substrates. (F) Secondary spectrum of the UBE3A protein. Co‐IP combined with western blotting was used to detect the protein‐protein interaction of VCPIP1 and UBE3A in HEK‐293T (G), DU145 (H) and LNCaP (I) cells. (J) The co‐localization of VCPIP1 and UBE3A within LNCaP and DU145 cells was assessed through immunofluorescence staining techniques. (K) The schematic representations of both the wild‐type and truncated mutants of VCPIP1, as well as UBE3A and its truncated mutants, were utilized in the subsequent Co‐IP assay. The results from the Co‐IP assays indicated that the OTU domain of VCPIP1 (L) and the C‐terminal region of UBE3A (M) are essential for their interaction. SPR analysis was performed to evaluate the direct binding between purified VCPIP1 and UBE3A. Representative kinetic fitting curves (N) and steady‐state affinity fitting curves (O) are shown.

To validate the findings from proteomics and mass spectrometry, we conducted co‐transfection of Flag‐VCPIP1 and Myc‐UBE3A into HEK‐293T cells. Co‐IP and western blot analyses demonstrated a specific exogenous interaction between Flag‐VCPIP1 and Myc‐UBE3A (Figure 4G). Additionally, the endogenous interaction between VCPIP1 and UBE3A was confirmed through Co‐IP and immunoblotting assays in LNCaP and DU145 cell lines (Figure 4H,I). Immunofluorescence assays further revealed the co‐localization of VCPIP1 and UBE3A proteins (Figure 4J).

To further elucidate the interaction between VCPIP1 and UBE3A, we engineered a series of truncated mutants for both proteins (Figure 4K). Specifically, three recombinant truncation constructs of VCPIP1 were developed, and subsequent Co‐IP assays indicated that the OTU domain of VCPIP1 is essential for its interaction with UBE3A (Figure 4L). Concurrently, two truncated constructs of UBE3A were generated: Myc‐UBE3A C (amino acids 451–875) and Myc‐UBE3A N (amino acids 1–450) (Figure 4K). Co‐IP analysis demonstrated that VCPIP1 interacted with both full‐length UBE3A and Myc‐UBE3A C, but not with Myc‐UBE3A N, suggesting that the C‐terminal region of UBE3A is critical for its binding to VCPIP1(Figure 4M). To further determine whether VCPIP1 directly interacts with UBE3A, we performed an in vitro SPR assay using purified proteins. SPR analysis revealed concentration‐dependent binding between VCPIP1 and UBE3A. Kinetic fitting yielded a dissociation constant (KD) of 1.09 × 10^−^^7^ M, while steady‐state affinity analysis yielded a comparable KD of 1.58 × 10^−^^7^ M (Figure 4N,O). These results provided direct biophysical evidence for the interaction between VCPIP1 and UBE3A.

Furthermore, altering the expression of VCPIP1 in LNCaP and DU145 cells did not affect UBE3A mRNA levels, as shown in Figure S3A. Nonetheless, knockdown of VCPIP1 led to a significant decrease in UBE3A protein levels, whereas overexpression of VCPIP1 resulted in a significant increase in UBE3A protein levels in these cells, as illustrated in Figure 5A,B. These findings suggested that VCPIP1 specifically interacts with UBE3A protein and regulates its expression at the post‐transcriptional level.

**FIGURE 5:**
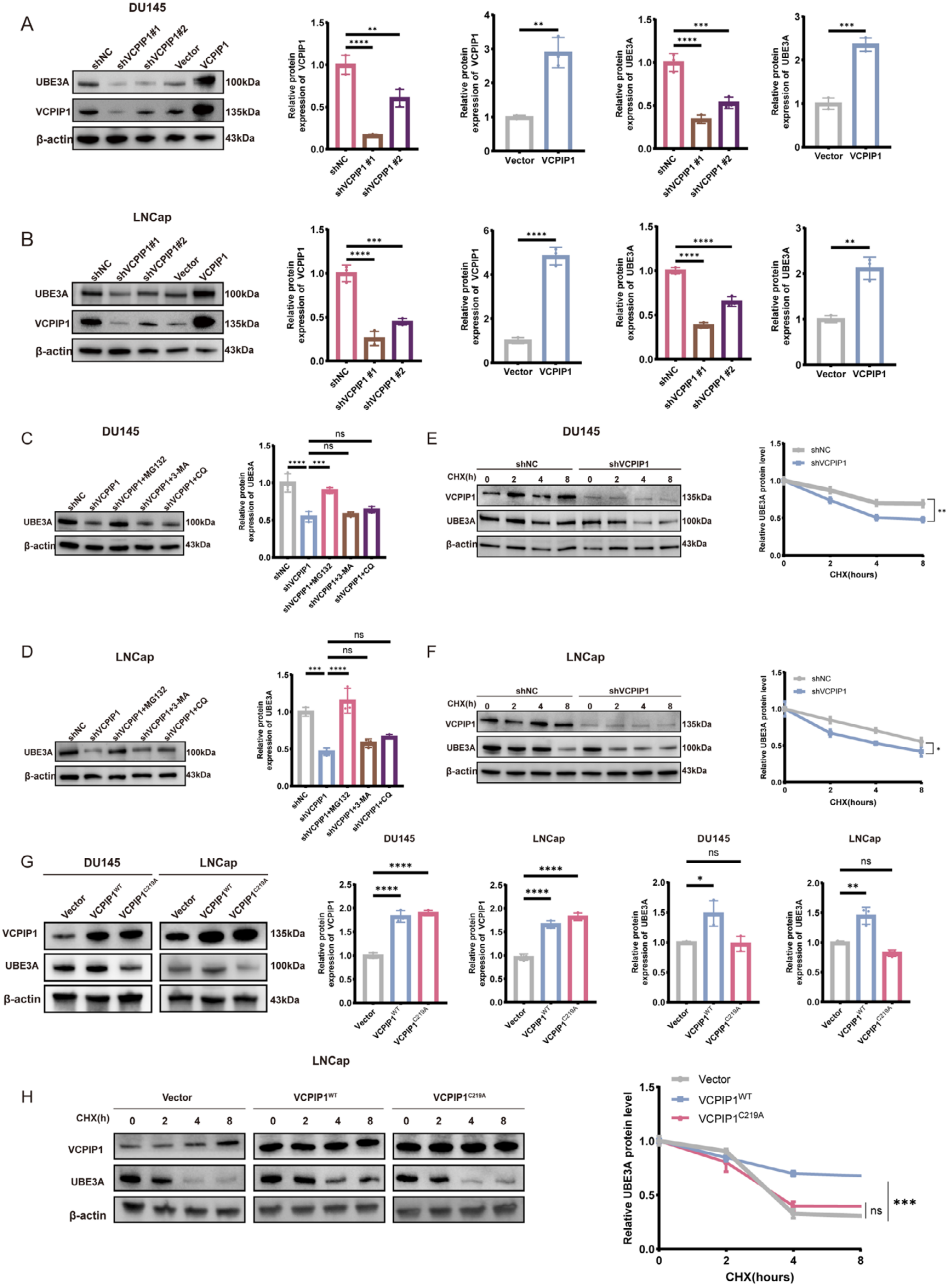
VCPIP1 stabilized UBE3A protein expression through its deubiquitinase activity. Western blot was used to detect the effect of VCPIP1 knockdown or overexpression on the protein expression levels of UBE3A in DU145 (A) and LNCaP (B) cells. Western blot analysis was performed to evaluate UBE3A protein expression in DU145 (C) and LNCaP (D) cells following treatment with MG132 (20 µM, 6 h), 3‐MA (10 mM, 6 h), or chloroquine (20 µM, 6 h). Cycloheximide (CHX) chase assays were performed to assess UBE3A protein stability in DU145 (E) and LNCaP (F) cells following VCPIP1 knockdown and treatment with CHX (50 µg/mL). (G) The protein levels of VCPIP1 and UBE3A in PCa cells after transfection with vector, VCPIP1 WT, or VCPIP1 C219A were measured via Western blotting. (H) The CHX protein half‐life assay was utilized to evaluate the stability and degradation rate of the UBE3A protein in LNCaP cells following transfection with either a vector, VCPIP1 WT, or VCPIP1 C219A mutant. Quantitative data are presented as the mean ± SD from three independent experiments. Statistical significance was assessed using an unpaired two‐tailed Student's *t*‐test for two‐group comparisons, one‐way ANOVA for multi‐group comparisons, and two‐way ANOVA for the CHX time‐course analyses. ^*^ *p *< 0.05, ^**^ *p *< 0.01, ^***^ *p *< 0.001, ^****^ *p *< 0.0001.

The ubiquitin‐proteasome pathway and the autophagy‐lysosome pathway constitute two fundamental systems for protein degradation. To examine the regulatory impact of VCPIP1 on UBE3A, DU145 and LNCaP cells with stable knockdown of VCPIP1 were treated with the proteasome inhibitor MG132, as well as the autophagy inhibitors 3‐methyladenine (3‐MA) and chloroquine. Western blot analysis demonstrated that MG132 treatment counteracted the VCPIP1 knockdown‐induced reduction in UBE3A expression, whereas the autophagy inhibitors did not significantly impact UBE3A protein levels in either cell line (Figure 5C,D). These findings indicated that VCPIP1 modulated UBE3A protein levels predominantly through a proteasome‐dependent degradation pathway. The results of cycloheximide (CHX) chase assay revealed that knockdown of VCPIP1 led to a prominent decrease in the stability of endogenous UBE3A protein in LNCaP and DU145 cells (Figure 5E,F).

We conducted an overexpression analysis of both the wild‐type and mutant (C219A) forms of VCPIP1 to investigate the necessity of its ubiquitin catalytic function for the expression and stability of UBE3A. Western blot analysis demonstrated that overexpression of VCPIP1 in LNCaP and DU145 cell lines resulted in elevated levels of UBE3A protein, whereas the mutant VCPIP1 did not produce similar effects (Figure 5G). Furthermore, the findings indicated that VCPIP1 overexpression contributed to the stabilization of the UBE3A protein, a result not observed with the mutant form of VCPIP1 (Figure 5H).

### 3.6 VCPIP1 Mediates the Deubiquitination of UBE3A

Subsequently, we explored the potential involvement of VCPIP1 in the removal of conjugated ubiquitin from the UBE3A protein. To clarify the deubiquitinating activity of VCPIP1 on UBE3A, we performed a deubiquitination assay. Results from an exogenous in vivo ubiquitination assay revealed a significant increase in the polyubiquitination levels of Myc‐UBE3A in HEK‐293T cells with VCPIP1 knockdown, while a notable decrease was observed in HEK‐293T cells transduced with Flag‐VCPIP1 (Figure 6A). Additionally, endogenous in vivo ubiquitination assays showed a substantial increase in the polyubiquitination levels of UBE3A in stable VCPIP1‐silenced LNCaP/DU145 cells and a significant decrease in stable VCPIP1‐overexpressing LNCaP/DU145 cells (Figure 6B,C). Moreover, additional assays demonstrated that the influence of VCPIP1 on UBE3A polyubiquitination is contingent upon the preservation of its catalytic activity, as the catalytically inactive variant of VCPIP1 (C219A) did not alter UBE3A polyubiquitination (Figure 6D).

**FIGURE 6:**
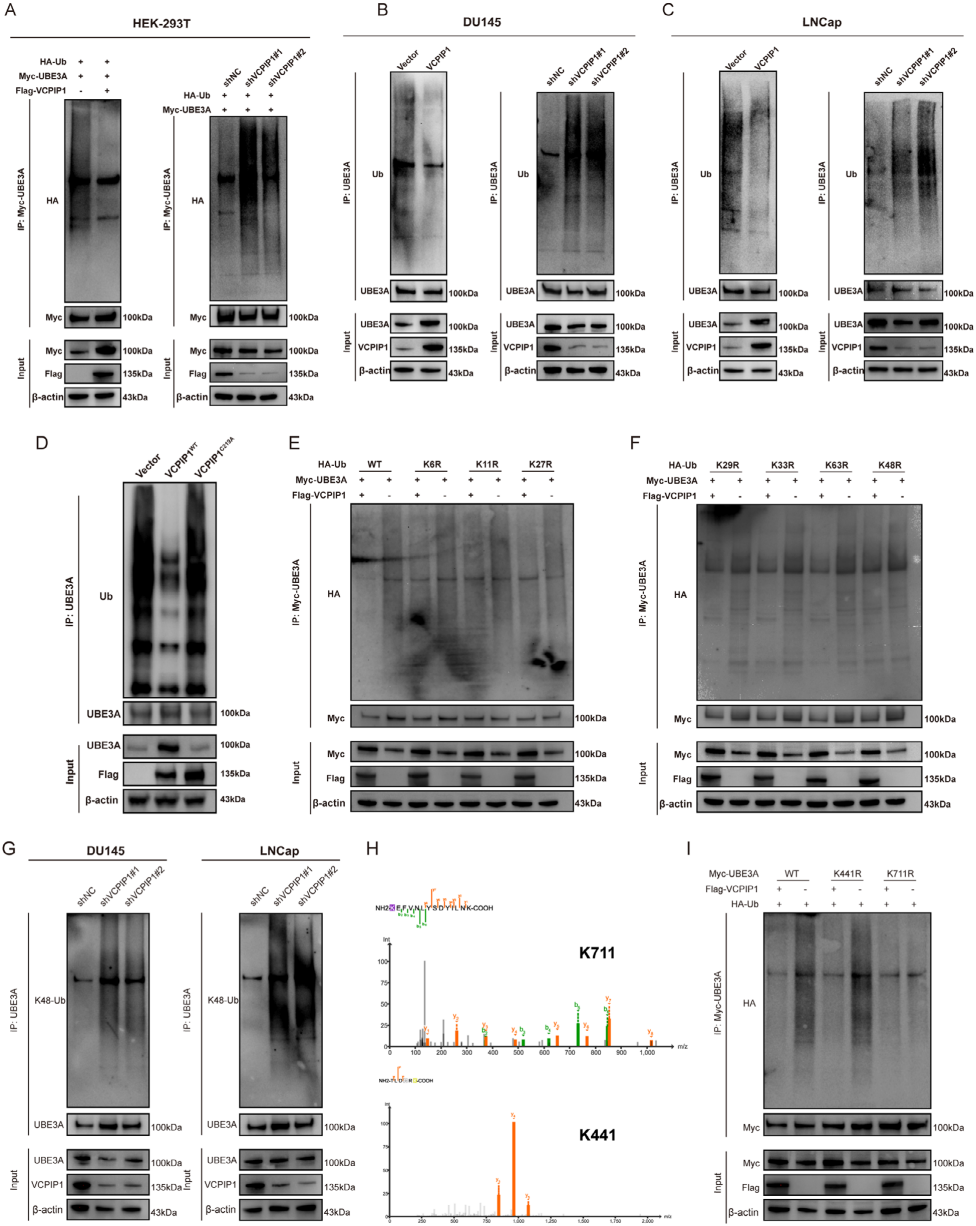
VCPIP1 removed K48‐linked ubiquitination of UBE3A at K711 in a deubiquitinase activity‐dependent manner. (A) Co‐IP combined with western blot analysis was employed to assess the impact of VCPIP1 overexpression or knockdown on the ubiquitination levels of exogenously expressed Myc‐UBE3A in HEK‐293T cells. Co‐IP combined with Western blot was used to detect the effect of VCPIP1 overexpression or knockdown on the ubiquitination levels of endogenous UBE3A in DU145 (B) and LNCaP (C) cells. (D) Co‐IP in conjunction with Western blot analysis was employed to assess the ubiquitination levels of UBE3A in LNCaP cells subsequent to transfection with either a control vector, wild‐type VCPIP1, or the VCPIP1 C219A mutant. (E,F) Seven distinct site‐directed mutagenesis plasmids of HA‐Ub were individually co‐transfected with the wild‐type Myc‐UBE3A into HEK‐293T cells. To assess the impact of mutations at various lysine residues in ubiquitin on the ubiquitination levels of exogenous Myc‐UBE3A, Co‐IP combined with Western blot analysis was employed. (G) Co‐IP combined with Western blot analysis was employed to assess the impact of VCPIP1 knockdown on the K48‐linked ubiquitination levels of endogenous UBE3A in DU145 and LNCaP cells. (H) Potential ubiquitination sites of UBE3A identified by mass spectrometry. (I) Lysine residues 441 and 711 of UBE3A were individually mutated to arginine to generate two distinct site‐directed mutant plasmids of UBE3A, each tagged with Myc. These Myc‐tagged UBE3A mutant plasmids were co‐transfected with HA‐Ub, Flag‐VCPIP1 into HEK‐293T cells. Subsequently, Co‐IP combined with western blot analysis was employed to assess the impact of mutations at the lysine 441 or 711 sites on the ubiquitination levels of exogenously expressed Myc‐UBE3A.

Ubiquitin is a protein consisting of 76 amino acids, featuring seven lysine residues (K6, K11, K27, K29, K33, K48, and K63), each of which can undergo ubiquitination to form distinct polyubiquitin chains. To determine which lysine residues of ubiquitin participate in the VCPIP1‐mediated deubiquitination of UBE3A, we substituted the lysine residues K6, K11, K27, K29, K33, K48, and K63 with arginine (resulting in K6R, K11R, K27R, K29R, K33R, K48R, and K63R mutations). We then co‐transfected Myc‐UBE3A, Flag‐VCPIP1, and HA‐Ub (including both wild‐type and mutant forms) into HEK‐293T cells. The results demonstrated that the deubiquitination effect of VCPIP1 overexpression on UBE3A was significantly diminished in the presence of the K48R mutant. In contrast, other mutant ubiquitins did not exhibit this effect, indicating that the K48 lysine residue on the polyubiquitin chain played a crucial role in the VCPIP1‐mediated deubiquitination of UBE3A, as illustrated in Figure 6E,F. An endogenous ubiquitination assay employing a K48‐linkage specific polyubiquitin antibody provided additional confirmation of the involvement of the K48 lysine residue in the VCPIP1‐mediated deubiquitination of UBE3A (Figure 6G).

Through the application of immunoprecipitation and mass spectrometry analysis, lysine residues at positions 441 and 711 were identified as potential ubiquitination sites in UBE3A (Figure 6H). To determine the involvement of the K441 (Lys‐441) and K711 (Lys‐711) lysine sites in the VCPIP1 deubiquitination process, we introduced mutations substituting lysine with arginine at these positions on UBE3A. Subsequently, we co‐transfected HEK‐293T cells with Myc‐UBE3A constructs (including wild‐type, K441R‐mutant, and K711R‐mutant), Flag‐VCPIP1, and HA‐Ub. The findings indicated that Lys‐711, rather than Lys‐441, was crucial in the VCPIP1‐mediated deubiquitination of UBE3A (Figure 6I).

### 3.7 UBE3A Promotes Malignant Biological Behaviors of PCa Cells

IHC staining was conducted to assess UBE3A protein expression in tissue samples obtained from the newly diagnosed mHSPC clinical cohort. The findings indicated a significantly elevated expression of UBE3A in cancerous tissues within the short CFS group compared to the long CFS group (Figure 7A,B). Correlation analysis identified a positive association between VCPIP1 and UBE3A protein expression in mHSPC tissues (Table 4 and Figure 7C). To further investigate the regulatory role of UBE3A in the malignant biological behavior of prostate cancer, stable DU145 and LNCaP prostate cancer cell lines with either UBE3A knockdown or overexpression were subsequently developed. The modified UBE3A expression levels were successfully validated (Figure S2A–D).

**FIGURE 7:**
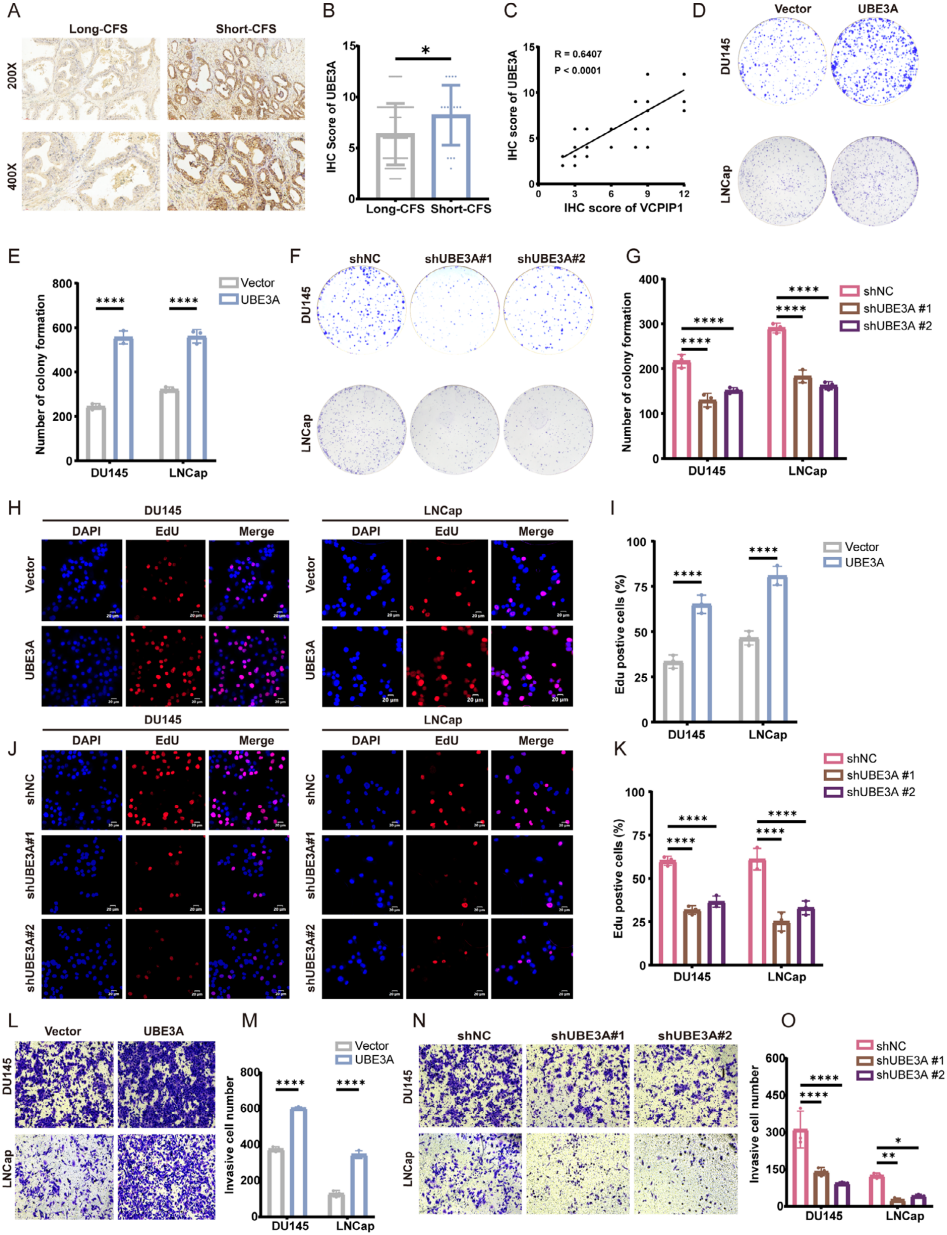
UBE3A promoted malignant biological behaviors of PCa cells. (A,B) IHC staining of UBE3A expression based on CFS stratification. Differences in UBE3A immunoreactivity scores between the short‐ and long‐CFS groups were analyzed using the Wilcoxon rank‐sum test. (C) The correlation between the expression levels of VCPIP1 and UBE3A in mHSPC tissues was assessed using Spearman's rank correlation analysis. The effect of overexpression (D,E) and knockdown (F,G) of UBE3A on the colony‐forming ability of DU145 and LNCaP cells was detected by colony formation assay. The effect of overexpression (H,I) and knockdown (J,K) of UBE3A on the proliferation of DU145 and LNCaP cells was detected by EdU assay. The effect of overexpression (L,M) and knockdown (N,O) of UBE3A on the invasion ability of DU145 and LNCaP cells was detected by the Transwell assay. Data are presented as the mean ± SD from three independent experiments. Statistical significance was assessed using unpaired two‐tailed Student's t‐test and one‐way ANOVA. ^*^ *p *< 0.05, ^**^ *p *< 0.01, ^****^ *p *< 0.0001.

**TABLE 4:** The expression levels of VCPIP1 and UBE3A in mHSPC tissues.

Group		VCPIP1 expression		Total	*p* value
		Low expression	High expression		
UBE3A expression	Low expression	36	12	48	*p *< 0.001
	High expression	10	70	80	
	Total	46	82	128	

The colony formation assay revealed that UBE3A overexpression significantly enhanced clonogenic efficiency in both DU145 and LNCaP cell lines, whereas UBE3A knockdown markedly inhibited cell proliferation (Figure 7D–G). The EdU assay demonstrated a significant increase in the proportion of EdU‐positive cells in the UBE3A‐overexpressing group compared to the empty vector control group, while UBE3A knockdown resulted in a marked reduction in the EdU‐positive cell ratio (Figure 7H–K). The Transwell assay indicated a positive correlation between UBE3A expression levels and cellular invasiveness. Specifically, UBE3A overexpression significantly enhanced the invasive capability of both DU145 and LNCaP cells, whereas UBE3A knockdown markedly inhibited their invasive potential (Figure 7L–O). Further analysis using the wound healing assay confirmed that UBE3A overexpression significantly promoted migratory activity in DU145 and LNCaP cells, whereas UBE3A knockdown substantially suppressed migration capacity (Figure 8A–D).

**FIGURE 8:**
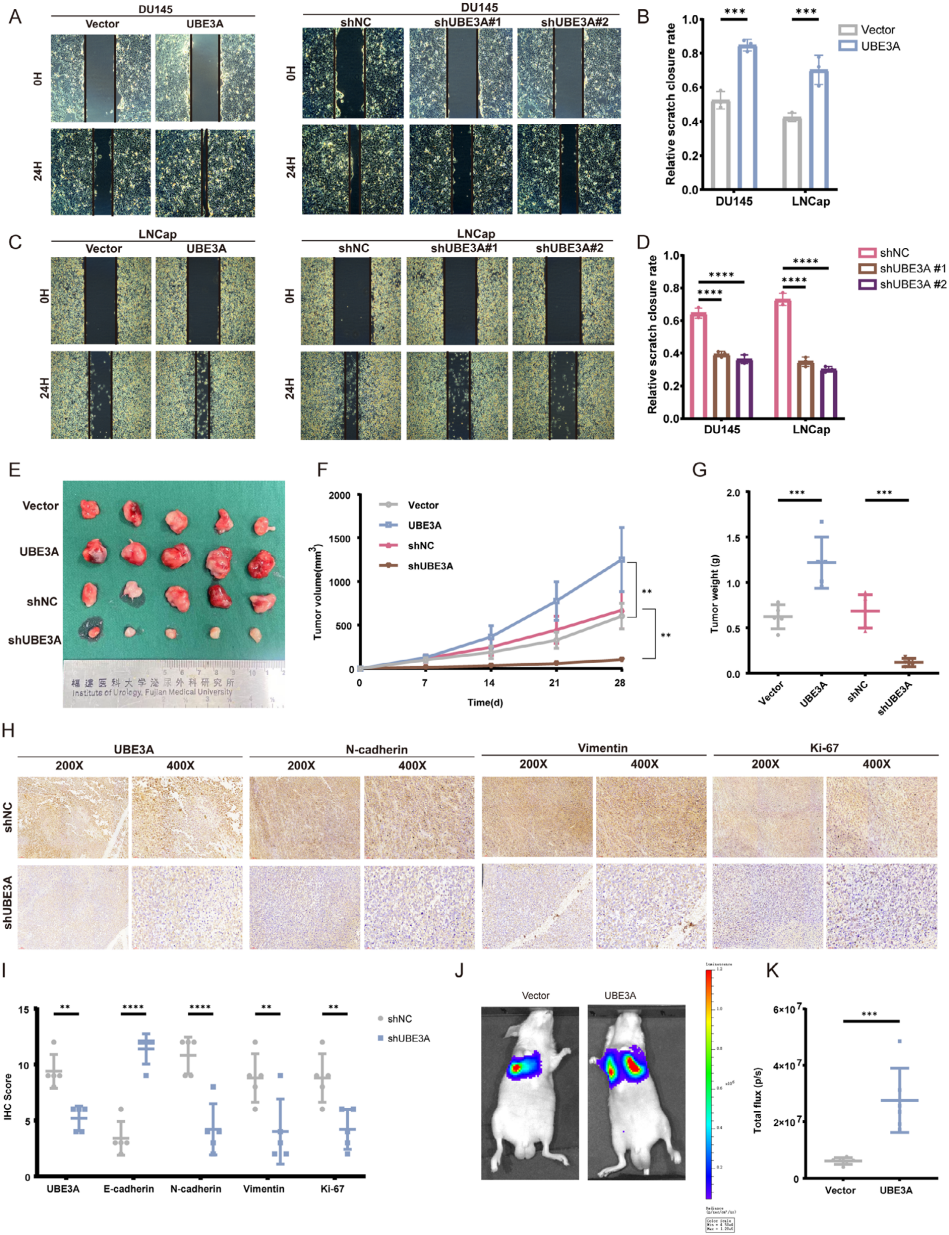
UBE3A promoted tumor formation and metastasis of prostate cancer in vivo. The effect of overexpression and knockdown of UBE3A on the migration ability of DU145 (A,B) and LNCaP (C,D) cells was detected by the scratch assay. (E–G) Different groups of DU145 cells (Vector, UBE3A, shNC, and shUBE3A) were subcutaneously inoculated in nude mice, *n* = 5. The tumor volume was measured every week and the growth curves were drawn. The tumor volume was calculated according to the formula: V = π/6×length×width^2^; after tumor cells injection 28 days, the nude mice were sacrificed to measure tumor weight. (H,I) IHC staining demonstrating the effects of UBE3A knockdown on EMT‐related protein markers and Ki67 proliferation index. Differences in immunohistochemical scores were analyzed using the Wilcoxon rank‐sum test. (J,K) In vivo bioluminescence imaging tracking metastatic dissemination of luciferase‐tagged DU145 cells (Vector vs. UBE3A) following tail vein injection, *n* = 6. Data are presented as the mean ± SD from three independent experiments. Statistical significance was assessed using an unpaired two‐tailed Student's *t*‐test for panels B, G, K, one‐way ANOVA for panel D, and two‐way repeated‐measures ANOVA for panel F. ^**^ *p *< 0.01, ^***^ *p *< 0.001, ^****^ *p *< 0.0001.

Experimental results from subcutaneous xenograft tumor models showed that UBE3A overexpression significantly increased tumor growth rate, volume, and weight compared to controls, while UBE3A knockdown inhibited these effects (Figure 8E–G). IHC staining demonstrated that a reduction in UBE3A expression led to a decrease in the mesenchymal markers N‐cadherin and Vimentin, as well as a reduction in Ki67‐positive proliferative cells (Figure 8H–I). Additionally, tail vein metastasis models demonstrated that UBE3A overexpression significantly enhanced metastatic activity in prostate cancer cells (Figure 8J–K).

### 3.8 VCPIP1 Downregulates p53 by Facilitating UBE3A‐Mediated Proteasomal Degradation

As an integral member of the HECT family of E3 ubiquitin ligases, UBE3A has been shown to play a crucial role in tumorigenesis by facilitating the polyubiquitination and subsequent proteasomal degradation of the p53 protein [21, 22]. To further explore the potential recognition and degradation of p53 by UBE3A in prostate cancer, we co‐transfected HEK‐293T cells with His‐tagged p53 and Myc‐UBE3A. Co‐IP and western blot analyses revealed an interaction between exogenously expressed Myc‐UBE3A and His‐p53 (Figure 9A). The endogenous interaction between UBE3A and p53 was also confirmed in both DU145 and LNCaP cells (Figure 9B,C). Additionally, immunofluorescence analysis further validated the co‐localization of UBE3A and p53 proteins (Figure 9E).

**FIGURE 9:**
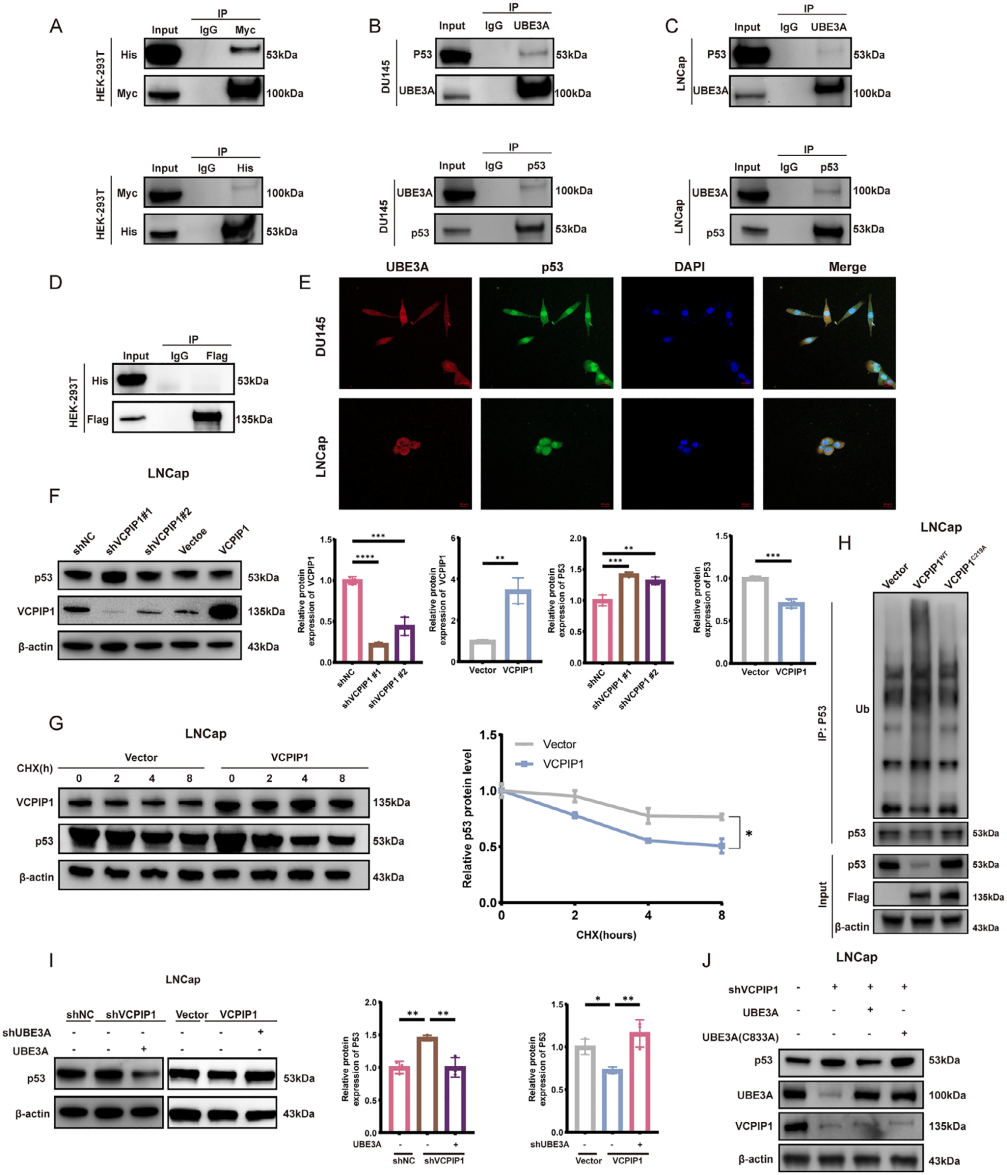
VCPIP1 promoted p53 ubiquitination and degradation via UBE3A in a deubiquitinase activity‐dependent manner. (A) Co‐IP combined with Western blot analysis was employed to investigate the interaction between exogenously expressed Myc‐UBE3A and His‐p53 proteins in HEK‐293T cells. (B) Co‐IP combined with western blot analysis was employed to investigate the interaction between endogenous UBE3A and p53 in DU145 cells. (C) Co‐IP combined with western blot analysis was employed to investigate the interaction between endogenous UBE3A and p53 in LNCaP cells. (D) Co‐IP combined with western blot analysis was employed to investigate the interaction between exogenously expressed Flag‐VCPIP1 and His‐p53 proteins in HEK‐293T cells. (E) The co‐localization of UBE3A and p53 in LNCaP and DU145 cells was examined through immunofluorescence staining. (F) Western blot was used to detect the effect of VCPIP1 knockdown or overexpression on the expression levels of p53 in LNCaP cells. (G) The CHX protein half‐life assay was employed to assess the impact of VCPIP1 overexpression on the stability and degradation rate of p53 protein in LNCaP cells. (H) Co‐IP in conjunction with Western blot analysis was employed to assess the ubiquitination levels of p53 in LNCaP cells subsequent to transfection with either a control vector, wild‐type VCPIP1, or the VCPIP1 C219A mutant. (I) UBE3A was overexpressed in LNCaP cells with stable knockdown of VCPIP1, whereas UBE3A was silenced in LNCaP cells exhibiting stable overexpression of VCPIP1. Subsequently, the expression levels of p53 in these modified cell lines were assessed via immunoblotting. (J) The ubiquitination activity of UBE3A is required for reversing VCPIP1 knockdown‑induced p53 upregulation, as wild‑type UBE3A, but not the C833A mutant, restored p53 expression. Quantitative data are presented as the mean ± SD from three independent experiments. Statistical significance was assessed using an unpaired two‐tailed Student's *t*‐test for two‐group comparisons, one‐way ANOVA for multi‐group comparisons, and two‐way ANOVA for the CHX time‐course analysis. ^*^ *p *< 0.05, ^**^ *p *< 0.01, ^***^ *p *< 0.001, ^****^ *p *< 0.0001.

Despite the absence of an observed interaction between VCPIP1 and p53, it was noted that knockdown of VCPIP1 resulted in an increased level of p53, whereas overexpression of VCPIP1 led to a reduction in p53 levels (Figure 9D,F and Figure S3B). The CHX chase assay further indicated that VCPIP1 overexpression significantly decreased the stability of endogenous p53 protein in LNCaP and DU145 cells (Figure 9G and Figure S3C). Additionally, further assays revealed that the effect of VCPIP1 on p53 polyubiquitination is dependent on its catalytic activity, as the catalytically inactive VCPIP1 mutant did not affect p53 polyubiquitination (Figure 9H). To investigate whether VCPIP1 modulated p53 levels via UBE3A, a series of experiments were conducted. These experiments involved UBE3A knockdown in DU145 and LNCaP cells overexpressing VCPIP1, as well as UBE3A transduction in DU145 and LNCaP cells with VCPIP1 knockdown. The results indicated that UBE3A knockdown increased p53 levels in DU145 and LNCaP cells overexpressing VCPIP1, whereas UBE3A transduction decreased p53 levels in cells with VCPIP1 knockdown (Figure 9I and Figure S3D). Considering that the ubiquitination activity of UBE3A is contingent upon a highly conserved cysteine residue at position 833, further experiments were undertaken to evaluate the significance of UBE3A's ubiquitination function in relation to VCPIP1‐mediated stabilization of the p53 protein. The findings indicated that wild‐type UBE3A, not the UBE3A‐C833A mutant, reversed the upregulated p53 expression caused by VCPIP1 knockdown (Figure 9J). These findings provided further evidence that VCPIP1 enhanced UBE3A expression through deubiquitination. This process, in turn, facilitated UBE3A‐mediated ubiquitin‐proteasomal degradation of p53, ultimately leading to reduced p53 expression in PCa cells.

### 3.9 UBE3A Ubiquitin Ligase Activity Is Required for VCPIP1‐Driven Malignant Phenotypes

To examine the role of VCPIP1 in facilitating the progression of prostate cancer through the upregulation of UBE3A, phenotypic rescue experiments were performed. EdU incorporation assays demonstrated that knockdown of VCPIP1 significantly decreased the proportion of proliferating cells. This reduction was completely reversed by the ectopic expression of wild‐type UBE3A, but not by the C833A mutant (Figure 10A,B). Similarly, colony formation assays indicated that the clonogenic potential of PCa cells was substantially diminished following VCPIP1 depletion, and this deficit was ameliorated exclusively by wild‐type UBE3A (Figure 10C,D). Wound‐healing assays further revealed that VCPIP1 knockdown inhibited cell migration, and the reintroduction of wild‐type UBE3A, but not the mutant variant, restored migratory capacity to levels comparable to those of control cells (Figure 10E,F). In alignment with these findings, Transwell invasion assays showed that VCPIP1 deficiency resulted in a significant decrease in the number of invasive cells, which was reversed by wild‐type UBE3A, whereas the mutant did not produce a notable effect (Figure 10G,H). Finally, tail vein metastasis models demonstrated that the knockdown of VCPIP1 significantly diminished the bioluminescent signal intensity of metastatic lung foci, suggesting a reduction in metastatic colonization. Notably, the overexpression of wild‐type UBE3A, as opposed to the C833A mutant, restored the metastatic potential of VCPIP1‐knockdown cells in vivo (Figure 10I,J). Taken together, these findings suggested that VCPIP1 advanced prostate cancer progression via UBE3A‐mediated ubiquitination activity.

**FIGURE 10:**
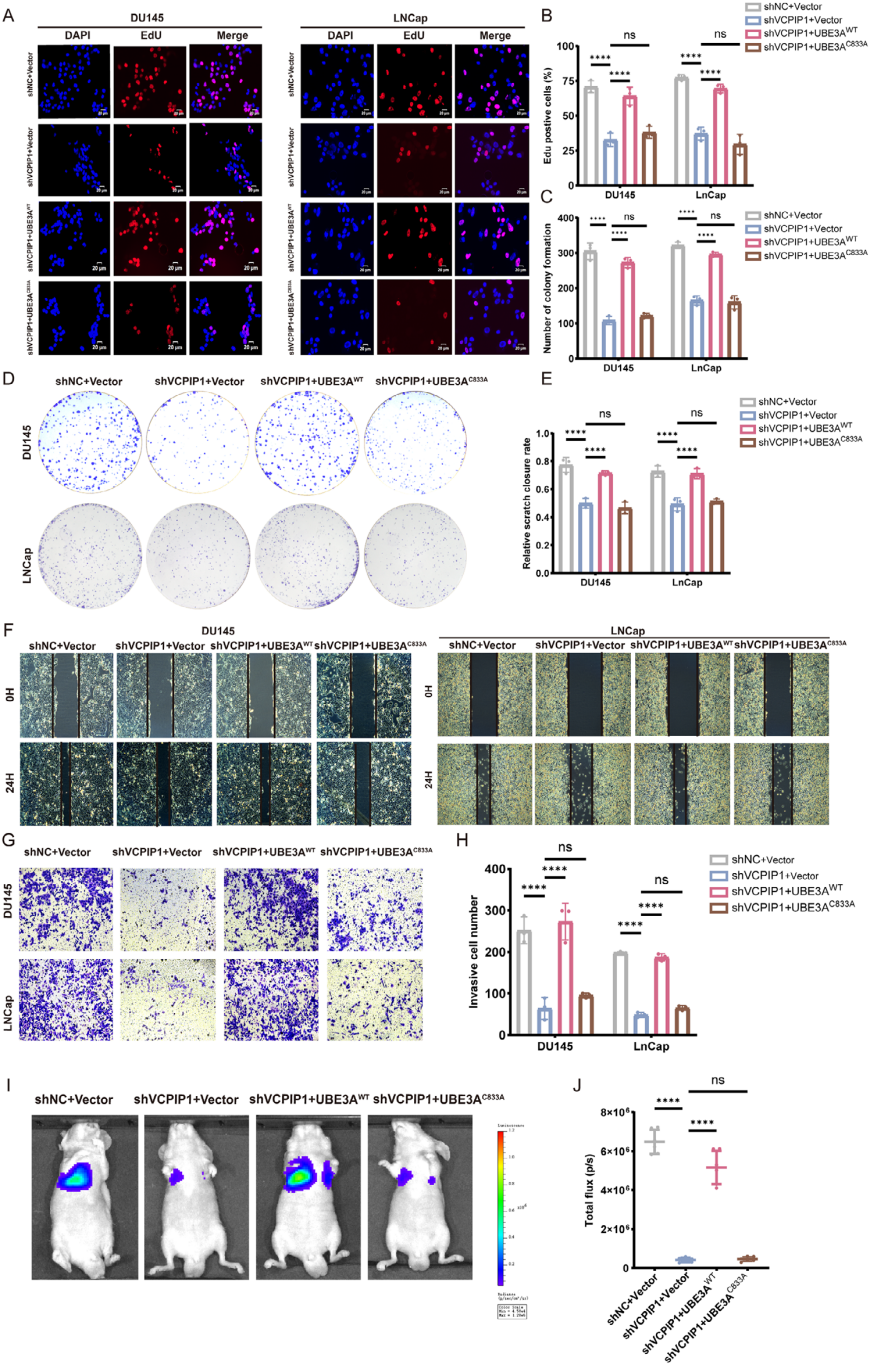
The ubiquitination activity of UBE3A is required for VCPIP1‐driven prostate cancer progression. (A,B) An EdU assay was employed to determine if the overexpression of UBE3A WT or UBE3A C833A could mitigate the impact of VCPIP1 knockdown on the reduced proliferative capacity of DU145 and LNCaP cells. (C,D) A colony formation assay was employed to evaluate whether the overexpression of UBE3A WT or UBE3A C833A could mitigate the impact of VCPIP1 knockdown on the reduced colony‐forming capacity of DU145 and LNCaP cells. (E,F) The scratch assay was employed to determine if the overexpression of UBE3A WT or UBE3A C833A could mitigate the impact of VCPIP1 knockdown on the reduced migratory capacity of DU145 and LNCaP cells. (G,H) The transwell assay was employed to assess whether the overexpression of UBE3A WT or UBE3A C833A could mitigate the impact of VCPIP1 knockdown on the reduced invasive capacity of DU145 and LNCaP cells. (I,J) The in vivo bioluminescence imaging was employed to determine if the overexpression of UBE3A WT or UBE3A C833A could mitigate the impact of VCPIP1 knockdown on the reduced metastatic dissemination of luciferase‐tagged DU145 cells following tail vein injection, *n* = 6. Data are presented as the mean ± SD from three independent experiments. Statistical significance was assessed using one‐way ANOVA. ^****^ *p *< 0.0001.

### 3.10 The Role of p53 in VCPIP1/UBE3A‐Driven Malignant Phenotypes Is TP53 Context‐Dependent

To further determine the functional contribution of p53 to VCPIP1/UBE3A‐driven malignant phenotypes, phenotypic rescue experiments were performed in prostate cancer cells with distinct TP53 backgrounds. In TP53‐wild‐type LNCaP cells, EdU incorporation and colony formation assays showed that VCPIP1 knockdown markedly suppressed cell proliferation and clonogenic capacity, whereas concomitant TP53 knockdown partially reversed these effects (Figure S4A–D). Similarly, the impaired migratory and invasive capacities induced by VCPIP1 depletion were partially restored following TP53 knockdown (Figure S4E–H). In contrast, in TP53‐mutant DU145 cells, concomitant TP53 depletion failed to rescue the inhibitory effects of VCPIP1 knockdown on proliferation, clonogenic growth, migration, and invasion (Figure S4A–H). These findings indicated that wild‐type p53 partially mediates the suppression of malignant phenotypes induced by VCPIP1 depletion in LNCaP cells, whereas the effects of the VCPIP1/UBE3A axis in DU145 cells are largely independent of mutant p53.

Given the central role of persistent AR signaling in prostate cancer progression, we next examined whether VCPIP1‐dependent regulation of wild‐type p53 is associated with AR expression in LNCaP cells. VCPIP1 knockdown significantly reduced both AR mRNA and protein levels, whereas concomitant TP53 knockdown substantially restored AR expression (Figure S4I–K). These findings indicated that restoration of wild‐type p53 contributes to the reduction in AR expression following VCPIP1 depletion.

### 3.11 Association of VCPIP1 Expression With Progression to CRPC

Utilizing hormone‐sensitive prostate cancer LNCaP cells, we developed subcutaneous HSPC tumor models. These models were subjected to surgical castration, and the recurrence of tumors under conditions of reduced testosterone levels was interpreted as the transition of HSPC models to CRPC. Post‐castration, we successfully established ten CRPC models (Figure 11A). Utilizing tumor tissues from various models, the expression of VCPIP1 was confirmed in HSPC and CRPC tissues through IHC staining and Western blot analysis. The findings revealed that VCPIP1 expression was significantly elevated in CRPC tissues compared to HSPC tissues (Figure 11B,C). Subsequently, three patient‐derived xenograft models of HSPC were established and subsequently transitioned into CRPC models through castration. The VCPIP1 expression observed in the HSPC and CRPC PDX models was consistent with that observed in our LNCaP CDX models (Figure 11D,E).

**FIGURE 11:**
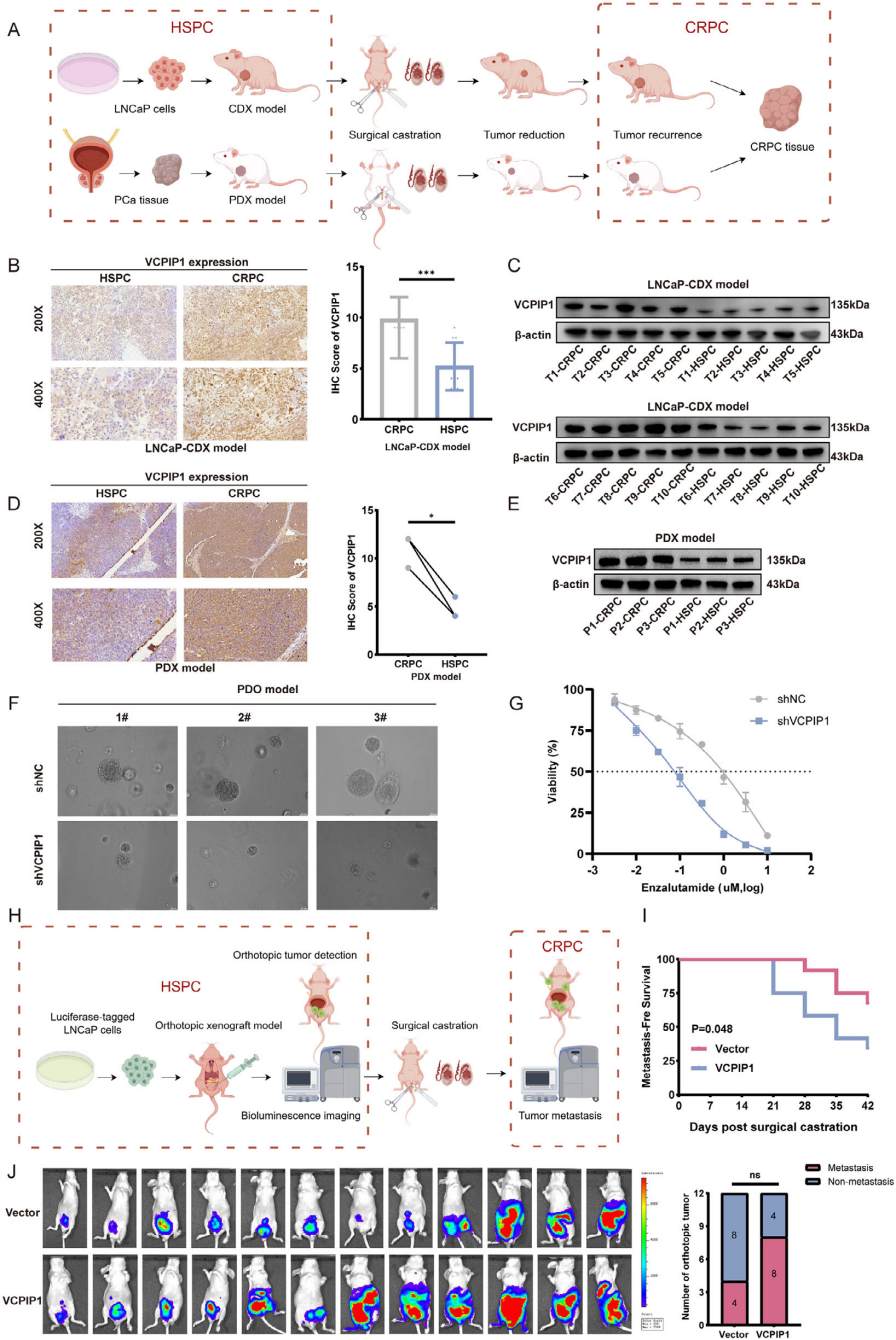
Association of VCPIP1 expression with progression to CRPC. (A) Surgical castration was conducted on mice with subcutaneous patient‐derived xenograft (PDX) or LNCaP cell‐derived xenograft (CDX) tumor tissues to induce and establish murine models of CRPC. (B) Representative IHC staining and quantitative analysis of VCPIP1 expression in HSPC and CRPC LNCaP CDX tissues, *n* = 10. Differences in VCPIP1 immunoreactivity scores between the HSPC and CRPC groups were analyzed using the Wilcoxon rank‐sum test. (C) Western blots for VCPIP1 expression levels in HSPC and CRPC LNCaP CDX models. (D) Representative IHC staining and matched‐pair analysis of VCPIP1 expression in three independent PDX models at the HSPC and CRPC stages, *n* = 3. Statistical significance was assessed using a paired two‐tailed Student's *t*‐test. (E) Western blot for VCPIP1 expression levels in HSPC and CRPC PDX models. (F) Representative images of prostate cancer patient‐derived organoids (PCa PDOs) after VCPIP1 knockdown. (G) The 3D CellTiter‐Glo assay was utilized to determine the half‐maximal inhibitory concentration (IC50) of enzalutamide in PCa PDOs subsequent to the knockdown of VCPIP1, *n* = 3. (H) Surgical castration was conducted on the orthotopic xenograft mouse model to induce and establish murine models of CRPC. (I) Kaplan‐Meier analysis of metastasis‐free survival in mice bearing orthotopic xenografts established with Vector‐ or VCPIP1‐overexpressing LNCaP cells. Metastasis‐free survival was compared using the log‐rank test. (J) In vivo bioluminescence imaging tracking metastatic dissemination of luciferase‐tagged LNCaP cells (Vector vs VCPIP1) following surgical castration, *n* = 12. Metastasis incidence was compared using Fisher's exact test. ^*^ *p *< 0.05, ^***^ *p *< 0.001.

Patient‐derived organoids, which closely resemble the genetic lineage and characteristics of in vivo tumors, including tissue architecture, cellular phenotype, heterogeneity, and drug responsiveness, have been successfully developed [23]. Enzalutamide, a second‐generation inhibitor of the androgen receptor, has shown efficacy in individuals diagnosed with metastatic prostate cancer [24]. To elucidate the role of VCPIP1 in modulating enzalutamide sensitivity within patient‐derived tissues, we conducted a depletion of VCPIP1 in organoids derived from in situ needle biopsies of prostate cancer. The findings demonstrated that VCPIP1 knockdown significantly enhanced the sensitivity of PDOs to enzalutamide, as evidenced by a reduced IC50, and was accompanied by a marked decrease in organoid size (Figure 11F,G).

To more accurately replicate an organ microenvironment that preserves the original tumor cell phenotypes, we inoculated stable VCPIP1‐overexpressing LNCaP cells and control cells into the dorsal prostate of Balb/c athymic nude mice to establish an orthotopic HSPC xenograft model. Following inoculation, these models underwent weekly bioluminescence imaging using an IVIS Spectrum to monitor orthotopic prostate tumorigenesis and any distant metastatic lesions. Tumor metastatic progression following castration was considered evidence of castration‐resistant progression (Figure 11H). Orthotopic prostate tumors became detectable as early as two weeks following inoculation, after which surgical castration was conducted. At six weeks post‐surgical castration, the incidence of tumor metastasis was 66.7% (8/12) in mice injected with VCPIP1‐overexpressing LNCaP cells, compared to 33.3% (4/12) in the control group (Figure 11J). The liver and femur were the most common sites of metastasis. Bone metastases were observed in three mice, one of which exhibited a pathological fracture. There was no statistically significant difference in the metastasis rate between the two groups (*p* = 0.22), which may be attributed to the follow‐up period being sufficiently long for the outcome to manifest. Survival analysis indicated that the VCPIP1‐overexpression orthotopic xenograft group exhibited significantly shorter metastasis‐free survival compared to the control group (Figure 11I). In conclusion, our findings indicated that VCPIP1 significantly contributed to the progression of CRPC.

## 4 Discussion

A substantial proportion of prostate cancer patients present with bone or visceral metastases at initial diagnosis, classified as mHSPC, which renders them ineligible for surgical intervention. The clinical management of these cases presents a significant challenge [25]. Although patients with newly diagnosed prostate cancer generally respond well to endocrine therapy, the majority ultimately advance to mCRPC within a few years [26]. Consequently, delaying the progression from mHSPC to mCRPC is a primary therapeutic objective in the management of metastatic prostate cancer [12]. Moreover, the pronounced heterogeneity of prostate cancer results in diverse tumor characteristics among patients, which leads to varied therapeutic responses to identical treatment regimens [27]. Notably, some patients experienced rapid progression from mHSPC to mCRPC shortly after diagnosis, exhibiting extremely poor prognoses. Therefore, developing a prognostic evaluation system based on molecular signatures to identify patients with rapidly progressive mHSPC at an early stage and implement stratified precision intervention strategies holds significant clinical value for enhancing patient quality of life [28, 29]. In this study, we utilized proteomic analysis and IHC staining to reveal a significantly increased expression of VCPIP1 in mCRPC and clinically rapid‐progressing mHSPC tissues. Survival analysis demonstrated that mHSPC patients exhibiting elevated VCPIP1 expression had a significantly shorter CRPC‐free survival compared to those with lower expression levels. Furthermore, multivariate Cox regression analysis identified the expression level of VCPIP1 as an independent prognostic factor for reduced CFS in mHSPC patients. Collectively, these findings suggested that VCPIP1 may play a critical regulatory role in the malignant progression from mHSPC to mCRPC.

VCPIP1 represents the largest deubiquitinating enzyme within the ovarian tumor protease (OTU) subfamily and plays a critical role in p97‐mediated Golgi membrane fusion following mitosis [30, 31]. Recent studies have further demonstrated that VCPIP1 exhibited multifaceted regulatory functions in tumor development [19, 32]. Li et al. [20] demonstrated that VCPIP1 interacted with and stabilized MATIIα in response to folic acid signaling, consequently facilitating the acceleration of the methionine cycle and contributing to the progression of hepatocellular carcinoma (HCC). Liu et al. [19] revealed that VCPIP1 stabilized YAP by preventing the K48‐linked polyubiquitination, thereby establishing a positive feedback loop with Hippo signaling and facilitating the progression of pancreatic adenocarcinoma (PAAD). Nevertheless, the function of VCPIP1 in the malignant progression of prostate cancer has not yet been elucidated. In this study, we undertook supplementary experiments to explore the functions of VCPIP1 in prostate cancer. Our results demonstrated that VCPIP1 facilitated multiple malignant phenotypes in PCa cells, such as proliferation, colony formation, invasion, and migration in vitro. These effects were critically reliant on its deubiquitinase activity, as evidenced by the inability of the catalytically inactive C219A mutant to induce these phenotypic changes. Subcutaneous tumor formation and tail vein transplantation experiments provided insights into the role of VCPIP1 in facilitating the growth and metastasis of prostate cancer. Furthermore, subcutaneous cell line‐derived xenograft and patient‐derived xenograft models were developed, revealing a significant upregulation of VCPIP1 expression in CRPC tissues compared to HSPC tissues. The findings from the orthotopic xenograft model demonstrated that the metastasis‐free survival in the VCPIP1 overexpression orthotopic xenograft cohort was markedly reduced relative to the control cohort. In patient‐derived organoids, VCPIP1 depletion enhanced sensitivity to enzalutamide and reduced organoid growth. These results collectively suggested that VCPIP1 played a pivotal role in facilitating the progression of CRPC via its deubiquitinase activity.

Protein homeostasis represents a highly intricate and interconnected physiological mechanism through which cells sustain protein expression, conformation, and subcellular localization [33, 34]. Disruptions in protein homeostasis can lead to pathological conditions [35]. Ubiquitination is a critical post‐translational modification that significantly impacts protein homeostasis and function [36]. This modification entails the covalent attachment of ubiquitin to target protein substrates, facilitated by a cascade of enzymes, thereby dictating the substrate's fate in terms of proteasomal degradation or signal transduction [37]. Conversely, deubiquitination serves as the reverse process, wherein deubiquitinating enzymes remove ubiquitin chains from substrates, thereby stabilizing target proteins and influencing various biological processes [38]. In this study, we employed quantitative proteomics and immunoprecipitation coupled with mass spectrometry analysis to elucidate the downstream molecular mechanisms of VCPIP1. Co‐IP and domain‐mapping experiments demonstrated that the OTU domain of VCPIP1 and the C‐terminal region of UBE3A are required for their interaction. Moreover, SPR analysis using purified proteins provided direct biophysical evidence for the binding between VCPIP1 and UBE3A. Subsequent experiments demonstrated that VCPIP1 stabilized UBE3A by removing K48‐linked polyubiquitin chains at Lys711. Functional rescue experiments further showed that wild‐type UBE3A, but not the catalytically inactive C833A mutant, restored the malignant phenotypes suppressed by VCPIP1 depletion, supporting UBE3A ubiquitin ligase activity as an important downstream mediator of VCPIP1 function. Consistently, both in vitro and in vivo experiments corroborated the tumor‐promoting role of UBE3A. Furthermore, our findings indicated that VCPIP1 facilitated prostate cancer progression specifically through UBE3A‐mediated ubiquitination activity.

As a pivotal regulator of genome stability, p53 fulfills its tumor‐suppressive role by precisely modulating key pathways such as cell cycle progression, DNA damage repair, and apoptosis [39]. The functional equilibrium of p53 is controlled by a complex regulatory network, with the ubiquitination modification system playing a crucial role in sustaining p53 activity through the dynamic regulation of its protein stability and subcellular localization [40]. Previous studies have demonstrated that the p53 protein serves as a ubiquitination substrate for UBE3A [21, 22]. In the tumorigenesis associated with HPV‐related malignancies, UBE3A interacted with the viral oncoprotein E6, forming a functional complex that facilitated the ubiquitination and subsequent degradation of p53 [41]. Zhang et al. [42] elucidated that the minichromosome maintenance protein MCM6 functioned as a molecular scaffold to recruit UBE3A, leading to the formation of an MCM6‐UBE3A‐p53 ternary complex, which promoted K48‐linked polyubiquitination of p53. Furthermore, Hou et al. [43] discovered that cinobufagin inhibited nasopharyngeal carcinoma metastasis by upregulating the tumor suppressor ENKUR. This upregulation stabilized p53 by inhibiting UBE3A‐mediated ubiquitination, thereby impeding epithelial‐mesenchymal transition. In this study, we further investigated the potential degradation of p53 via the UBE3A‐mediated ubiquitin‐proteasome pathway in prostate cancer. Given that TP53 gene mutations have been identified in numerous human cancers, it is noteworthy that while the incidence of TP53 mutations is relatively low in the initial stages of prostate cancer, the mutation rate escalates markedly as the disease advances [44, 45, 46]. Interestingly, our study demonstrated that UBE3A interacted with p53 in both hormone‐sensitive LNCaP cells, which express wild‐type p53, and hormone‐resistant DU145 cells, which express mutant p53. Moreover, VCPIP1 was found to reduce p53 expression by upregulating UBE3A expression in prostate cancer cells. These findings further substantiated the significant regulatory role of the VCPIP1/UBE3A axis in modulating p53 expression in prostate cancer. Importantly, our findings further demonstrated that the functional contribution of p53 to VCPIP1/UBE3A‐driven malignant phenotypes is dependent on the TP53 genetic context. In TP53‐wild‐type LNCaP cells, concomitant TP53 depletion partially rescued the impaired proliferation, clonogenic growth, migration, and invasion caused by VCPIP1 knockdown, indicating that restoration of wild‐type p53 represents an important mediator of the phenotypic effects induced by VCPIP1 depletion. In contrast, TP53 depletion failed to rescue these phenotypes in TP53‐mutant DU145 cells, indicating that the tumor‐promoting activity of the VCPIP1/UBE3A axis in this cellular context is largely independent of mutant p53. These observations suggest that additional UBE3A substrates and other p53‐independent pathways may contribute to the oncogenic effects of VCPIP1 in TP53‐mutant prostate cancer. Persistent AR signaling represents a central mechanism underlying prostate cancer progression and the development of castration resistance. A previous study demonstrated that wild‐type p53 negatively regulates AR transcription in LNCaP cells, with ChIP analysis showing p53 association with the 5^,^ regulatory region of the AR gene [47]. In AR‐positive, TP53‐wild‐type LNCaP cells, VCPIP1 depletion reduced both AR mRNA and protein expression, whereas concomitant TP53 depletion substantially restored AR expression. These findings suggested that VCPIP1 may help sustain AR expression by attenuating wild‐type p53 activity. However, the present experiments evaluated AR expression rather than AR transcriptional activity, subcellular localization, or downstream target‐gene activation, and were restricted to LNCaP cells. Therefore, whether VCPIP1 directly modulates AR signaling remains to be further investigated.

In conclusion, this study identified a VCPIP1/UBE3A deubiquitination–ubiquitination cascade that contributes to prostate cancer progression and the transition to CRPC. Wild‐type p53 represents an important downstream mediator of this pathway, whereas p53‐independent mechanisms appear to predominate in the TP53‐mutant context. These findings provide new mechanistic insights into CRPC progression and support further investigation of VCPIP1 as a potential prognostic biomarker and therapeutic vulnerability. Further preclinical studies are required to determine its therapeutic tractability and translational potential.

## Author Contributions

Writing the original Draft: Fei Lin, Wen‐Cai Zheng, Yi‐Qiu Wang, Bin Lin. Writing, review & Editing: Ning Xu and Yong Wei. Methodology: Fei Lin and Wen‐Cai Zheng. Investigation: Bin Lin and Xiao‐Dong Li. Formal Analysis: Wen‐Cai Zheng and Yi‐Qiu Wang. Data Curation: Yun‐Zhi Lin, Dong‐Ning Chen, Conceptualization: Fei Lin. Supervision: Xue‐Yi Xue and Yong Wei. Project administration: Ning Xu and Xue‐Yi Xue.

## Ethical Statement

All experiments were conducted in accordance with the relevant institutional and national guidelines and regulations of China. The study protocol was reviewed and approved by the Ethics Committee of the First Affiliated Hospital of Fujian Medical University (Approval No. MTCA, ECFAH of FMU [2024]432). All animal experiments were approved by the Experimental Animal Ethics Committee of Fujian Medical University (Approval No. IACUC FJMU 2024‐Y‐1724).

## Consent

Informed consent for publication of the manuscript was obtained from all authors.

## Conflicts of Interest

The authors declare no conflicts of interest.

## Supporting information




**Supporting File 1**: advs78433‐sup‐0001‐SuppMat.docx.

**Supporting File 2**: advs78433‐sup‐0002‐Tables.zip.

## Data Availability

The data that support the findings of this study are available from the corresponding author upon reasonable request.
