# Supplementary material for: The VCPIP1‐UBE3A Deubiquitination‐Ubiquitination Cascade Contributes to Castration‐Resistant Prostate Cancer Progression
Source: Adv Sci (Weinh). 2026 Oct 25:e78433. Online ahead of print. doi: 10.1002/advs.78433 (PMC13696778; doi:10.1002/advs.78433)
Supplement: Supplementary file 1 [file ADVS-9999-e78433-s002.docx]

**Figure S1. Validation of VCPIP1 overexpression and knockdown efficiency in DU145 and LNCaP cells.**

The qRT-PCR **(A)** and Western blot analysis **(B)** were employed to assess the expression levels of VCPIP1 in RWPE-1, DU145, and LNCaP cell lines. The qRT-PCR **(C-D)** and Western blot analysis **(E-F)** were employed to assess the overexpression efficiency of VCPIP1 in DU145 and LNCaP cell lines. The qRT-PCR **(G-H)** and Western blot analysis **(I-J)** were employed to assess the knockdown efficiency of VCPIP1 in DU145 and LNCaP cell lines. Data are presented as the mean ± SD from three independent experiments. Statistical significance was assessed using one-way ANOVA. **P*<0.05, ****P*<0.001, *****P*<0.0001.

**Figure S2. Validation of UBE3A overexpression and knockdown efficiency in DU145 and LNCaP cells.**

**(A)** The qRT-PCR was employed to assess the overexpression efficiency of UBE3A in DU145 and LNCaP cell lines. **(B)** The qRT-PCR was employed to assess the knockdown efficiency of UBE3A in DU145 and LNCaP cell lines. **(C)** Western blot analysis was utilized to evaluate the efficiency of UBE3A overexpression and knockdown in DU145 cells. **(D)** Western blot analysis was utilized to evaluate the efficiency of UBE3A overexpression and knockdown in LNCaP cells. Data are presented as the mean ± SD from three independent experiments. Statistical significance was assessed using unpaired two-tailed Student’s t-test and one-way ANOVA. ****P*<0.001, *****P*<0.0001.

**Figure S3. VCPIP1 promoted p53 degradation through UBE3A in DU145 cells.**

**(A)** qRT-PCR was used to detect the effect of VCPIP1 knockdown or overexpression on the mRNA expression levels of UBE3A in DU145 and LNCaP cells. **(B)** Western blot was used to detect the effect of VCPIP1 knockdown or overexpression on the expression levels of p53 in DU145 cells. **(C)** The CHX protein half-life assay was employed to assess the impact of VCPIP1 overexpression on the stability and degradation rate of p53 protein in DU145 cells. **(D)** UBE3A was overexpressed in DU145 cells with stable knockdown of VCPIP1, whereas UBE3A was silenced in DU145 cells exhibiting stable overexpression of VCPIP1. Subsequently, the expression levels of p53 in these modified cell lines were assessed via immunoblotting. Quantitative data are presented as the mean ± SD from three independent experiments. Statistical significance was assessed using an unpaired two-tailed Student’s t-test for two-group comparisons, one-way ANOVA for multi-group comparisons, and two-way ANOVA for the CHX time-course analysis. **P*<0.05, ***P*<0.01, ****P*<0.001, *****P*<0.0001.

**Figure S4. The contribution of p53 to VCPIP1/UBE3A-driven malignant phenotypes is TP53 context-dependent.**

**(A-B)** EdU incorporation assays were performed to assess whether TP53 knockdown could rescue the reduced proliferative capacity induced by VCPIP1 depletion in TP53-wild-type LNCaP and TP53-mutant DU145 cells. **(C-D)** Colony formation assays were performed to evaluate whether TP53 knockdown could rescue the impaired clonogenic capacity caused by VCPIP1 depletion in LNCaP and DU145 cells. **(E-F)** Transwell assays were performed to evaluate the effects of concomitant TP53 knockdown on the impaired invasive capacity caused by VCPIP1 depletion in LNCaP and DU145 cells. **(G-H)** Wound-healing assays were performed to assess the effects of concomitant TP53 knockdown on the reduced migratory capacity induced by VCPIP1 depletion in LNCaP and DU145 cells. **(I-K)** qRT-PCR and Western blot analyses were performed to assess AR mRNA and protein expression in LNCaP cells following VCPIP1 knockdown with or without concomitant TP53 depletion. Quantitative data are presented as the mean ± SD from three independent experiments. Statistical significance was assessed using one-way ANOVA. *P < 0.05, **P < 0.01, ***P < 0.001, and ****P < 0.0001.
