# Supplementary material for: The VCPIP1‐UBE3A Deubiquitination‐Ubiquitination Cascade Contributes to Castration‐Resistant Prostate Cancer Progression
Source: Adv Sci (Weinh). 2026 Oct 25:e78433. Online ahead of print. doi: 10.1002/advs.78433 (PMC13696778; doi:10.1002/advs.78433)
Supplement: Supplementary file 2 [file ADVS-9999-e78433-s001.zip › advs78433-sup-0003-Table2.docx]

| **Supplemental Table 2. Sequences of primers used for RT-qPCR.** | |
| --- | --- |
| **Genes** | **Sequences** |
| **VCPIP1** |  |
| F | 5’- TGTCAGCATCGAGTGTACCG -3’ |
| R | 5’- AGTTGGAAAGGCCCATCACC -3’ |
| **UBE3A** |  |
| F | 5’-CCCTGATGATGTGTCTGTGG-3’ |
| R | 5’-GGCAAAGCCATTTCCAGATA-3’ |
| **GAPDH** |  |
| F | 5’-GGTGTGAACCATGAGAAGTATGA-3’ |
| R | 5’-GAGTCCTTCCACGATACCAAAG-3’ |
| **AR** |  |
| F | 5’-TGGATGGCTCCAAATCACCC-3’ |
| R | 5’-AAGCGTCTTGAGCAGGATGT-3’ |
