# Supplementary material for: The VCPIP1‐UBE3A Deubiquitination‐Ubiquitination Cascade Contributes to Castration‐Resistant Prostate Cancer Progression
Source: Adv Sci (Weinh). 2026 Oct 25:e78433. Online ahead of print. doi: 10.1002/advs.78433 (PMC13696778; doi:10.1002/advs.78433)
Supplement: Supplementary file 2 [file ADVS-9999-e78433-s001.zip › advs78433-sup-0004-Table3.docx]

| **Supplemental Table 3. Top 20 upregulated proteins in the short-CFS versus long-CFS group among the 79 overlapping candidates.** | | | | |
| --- | --- | --- | --- | --- |
| **Protein accession** | **Protein description** | **Gene name** | **short-CFS/long-CFS Ratio** | **short-CFS/long-CFS P value** |
| Q58DX5 | Inactive N-acetylated-alpha-linked acidic dipeptidase-like protein 2 OS=Homo sapiens OX=9606 GN=NAALADL2 PE=1 SV=3 | NAALADL2 | 10.4444607 | 0.048521409 |
| Q99996 | A-kinase anchor protein 9 OS=Homo sapiens OX=9606 GN=AKAP9 PE=1 SV=4 | AKAP9 | 6.500906349 | 0.002060709 |
| Q9UHK6 | Alpha-methylacyl-CoA racemase OS=Homo sapiens OX=9606 GN=AMACR PE=1 SV=2 | AMACR | 6.1335165 | 0.040834404 |
| Q86YH6 | All trans-polyprenyl-diphosphate synthase PDSS2 OS=Homo sapiens OX=9606 GN=PDSS2 PE=1 SV=2 | PDSS2 | 5.609943363 | 0.040424136 |
| P07919 | Cytochrome b-c1 complex subunit 6, mitochondrial OS=Homo sapiens OX=9606 GN=UQCRH PE=1 SV=2 | UQCRH | 4.872528321 | 0.004727422 |
| P43897 | Elongation factor Ts, mitochondrial OS=Homo sapiens OX=9606 GN=TSFM PE=1 SV=2 | TSFM | 4.720363497 | 0.000229523 |
| Q9NR30 | Nucleolar RNA helicase 2 OS=Homo sapiens OX=9606 GN=DDX21 PE=1 SV=5 | DDX21 | 4.654328505 | 0.013520923 |
| Q96JH7 | Deubiquitinating protein VCPIP1 OS=Homo sapiens OX=9606 GN=VCPIP1 PE=1 SV=2 | VCPIP1 | 4.60346653 | 0.013541224 |
| P80723 | Brain acid soluble protein 1 OS=Homo sapiens OX=9606 GN=BASP1 PE=1 SV=2 | BASP1 | 4.538154858 | 0.006204083 |
| Q92922 | SWI/SNF complex subunit SMARCC1 OS=Homo sapiens OX=9606 GN=SMARCC1 PE=1 SV=3 | SMARCC1 | 4.381017522 | 0.048189705 |
| Q9BTY7 | Protein HGH1 homolog OS=Homo sapiens OX=9606 GN=HGH1 PE=1 SV=1 | HGH1 | 4.340052678 | 0.016757792 |
| Q6PI48 | Aspartate--tRNA ligase, mitochondrial OS=Homo sapiens OX=9606 GN=DARS2 PE=1 SV=1 | DARS2 | 4.136204101 | 0.000736937 |
| Q9NQH7 | Xaa-Pro aminopeptidase 3 OS=Homo sapiens OX=9606 GN=XPNPEP3 PE=1 SV=1 | XPNPEP3 | 4.11784428 | 0.04236241 |
| Q8NFV4 | Protein ABHD11 OS=Homo sapiens OX=9606 GN=ABHD11 PE=1 SV=1 | ABHD11 | 3.976279455 | 0.020941461 |
| Q96GX2 | Ataxin-7-like protein 3B OS=Homo sapiens OX=9606 GN=ATXN7L3B PE=1 SV=2 | ATXN7L3B | 3.950820939 | 0.038949958 |
| Q8IY81 | pre-rRNA 2'-O-ribose RNA methyltransferase FTSJ3 OS=Homo sapiens OX=9606 GN=FTSJ3 PE=1 SV=2 | FTSJ3 | 3.883854338 | 0.035111707 |
| P78330 | Phosphoserine phosphatase OS=Homo sapiens OX=9606 GN=PSPH PE=1 SV=2 | PSPH | 3.810180982 | 0.021030811 |
| Q9HCC0 | Methylcrotonoyl-CoA carboxylase beta chain, mitochondrial OS=Homo sapiens OX=9606 GN=MCCC2 PE=1 SV=1 | MCCC2 | 3.787562875 | 0.00615052 |
| Q9NWU1 | 3-oxoacyl-[acyl-carrier-protein] synthase, mitochondrial OS=Homo sapiens OX=9606 GN=OXSM PE=1 SV=1 | OXSM | 3.631848518 | 0.028539648 |
| P82932 | 28S ribosomal protein S6, mitochondrial OS=Homo sapiens OX=9606 GN=MRPS6 PE=1 SV=3 | MRPS6 | 3.425567862 | 0.017859875 |
